# Qualified placebo for trials of herbal medicine treatment in rare diseases? A cross-sectional analysis

**DOI:** 10.1186/s13023-023-02987-w

**Published:** 2023-11-30

**Authors:** Yixuan Li, Peipei Du, Xuebin Zhang, Chenyu Ren, Xinyi Shi, Xinglu Dong, Chi Zhang

**Affiliations:** 1 Dongzhimen Hospital, Beijing University of Chinese Medicine, 5 Haiyuncang Street, Dongcheng District, Beijing, 100070 China; 2 Institute for Brain Disorders, Beijing University of Chinese Medicine, Beijing, China

**Keywords:** Herbal medicine, Rare disease, Trials, Placebo, Cross-sectional analysis

## Abstract

**Background:**

While substantial placebos have been used in herbal medicine (HM) clinical trials for rare diseases, the use and quality of reporting of HM-placebo remain unclear. We aim to describe the use of HM-placebo in clinical trials for rare diseases and determine the quality of reporting in these trials.

**Methods:**

This is a cross-sectional study. We searched PubMed, Embase, Web of Science, SinoMed, China National Knowledge Infrastructure, WanFang database, China Science and Technology Journal Database, National Institute of Informatics Support Academic Information Services, ClinicalTrials.gov and Chinese Clinical Trials Registry from their inception date to 14 February 2023 to identify registered and published trials that use placebos as a comparator in rare diseases. We collected data on placebo use reporting and the efficacy and safety of placebo. Descriptive statistics, the Chi-square test, and Binary multivariable logistic regression analysis were used to determine the placebo characteristics of the HM trial and its effect on reporting.

**Results:**

Among the 55 studies, we included that with a median administration time of placebo of 84 days (IQR 42–180) and a median placebo sample size of 30 (IQR 24–54). About half of the trials (27, 49.1%) did not provide their ethical approvals, and one trial had details of informed consent. None of the studies were fully reported and more than half of the items reported less than 50%. A total of 10 trials (18.2%) of placebo has active ingredients even though none of them performed pharmacological inert tests. Of the 29 studies with available data on adverse events, 5 (17.2%) trials did not show a better safety profile in the placebo group. Under the context that a relatively high-quality report is defined as a report with more than 9 items, there was a statistically significant difference between the two groups in the rate of relatively high-quality reports of the administration time (*p* = 0.047, OR 0.10, 95% CI 0.01 to 0.90), but the results are not representative.

**Conclusion:**

The overall situation of HM-placebo in the field of rare diseases was poor. In particular, the placebo is tied to the quality of trials, and poor placebo hinders the generation of high-quality evidence for herbal clinical trials in the field of rare diseases. We summarize the current methods of assessment involved in the use of placebos and propose various considerations for placebos in different contexts. Our study can greatly promote rare disease researchers to review the quality of their placebo and clinical trials. It is imperative to guarantee that meticulously conducted research generates clinical evidence of the highest caliber. We also expect that in the future, more rigorous relevant standards about the reporting and design of HM-placebo will be developed. High-quality clinical trials are the prerequisite for the wide clinical application of herbal medicines for rare diseases.

**Supplementary Information:**

The online version contains supplementary material available at 10.1186/s13023-023-02987-w.

## Introduction

Rare diseases are a group of diseases that are uncommon and have a very low prevalence compared to other diseases [1]. Currently, there are approximately 7000 different rare diseases affecting 3.5–5.9% of individuals worldwide, which amounts to 263–446 million individuals [2]. There are many kinds of rare diseases with complex etiology. Currently, the rare disease lists of different countries do not contain exactly the same diseases. The terms “rare disease” and “orphan drug” are used most widely and the average prevalence threshold is between 40 and 50 cases/100,000 people. As a result, the terminology and prevalence thresholds used to define rare diseases vary across jurisdictions and organizations [3]. At the same time, only 10% of rare diseases in the world are treated accordingly, and ~ 7000 rare diseases still lack specific treatments [4, 5]. The cure of rare diseases and the research of new drugs for rare diseases are common expectations of researchers worldwide.

Herbal medicine (HM), one of the main treatment modalities in the world, has a history of thousands of years and is still actively used in Asia and elsewhere worldwide. In modern times, HM is widely used in rare diseases, and experimental evidence has been obtained. Through our preliminary search in Web of Science, CNKI, and other databases on February 14, 2023. A total of 528 clinical trials of HM were found to have been conducted in the rare disease field. Among these clinical trials, there were also well-designed trials with effective for therapeutics such as ImmunoGuard^®^, which showed safety and efficacy for the management of patients with Familial Mediterranean Fever [6]. However, some published systematic reviews of HM trials in rare diseases suggest that the quality of clinical studies with poor trial designs still needs to be improved. Placebo-controlled trials were included [7–9].

For clinical trials of drugs, it is essential to follow the basic principles of randomization, repetition, blindness, and control [10]. High-quality randomized, double-blind, and placebo-controlled clinical trials can provide evidence to support HM treatment approaches [11]. Criticism of the quality of HM-placebos used is unfortunately common. The preparation and use of HM-placebos have been continuously explored by researchers [10, 12]. However, due to the characteristics of rare disease clinical trials, the use of HM-placebos in rare diseases has not been analyzed.

Given the critical need to generate robust clinical evidence, it is essential that clinical trials are completed with high quality. Our objective was to systematically review clinical trials that had been conducted in the field of rare diseases and to collect relevant trial information, with a focus on the use of placebo in the trials and on placebo reporting. This study accurately captures the problems in the field of herbal medicine in the treatment of rare diseases and provides references for future researchers to conduct safer and more effective clinical trials.

## Methods

### Data source

We conducted a cross-sectional analysis of HM clinical trials focusing on rare diseases. We searched 8 electronic databases, including PubMed, Embase, Web of Science, SinoMed, China National Knowledge Infrastructure (CNKI), WanFang database, China Science and Technology Journal Database (VIP), and National Institute of Informatics Support Academic Information Services (CiNii). The literature retrieval time ranged from the inception of each database to 14 February 2023. Ongoing trials and unpublished studies were searched via ClinicalTrials.gov and the Chinese Clinical Trials Registry (ChiCTR). The retrieval strategies are listed in Additional file 1.

### Selection criteria

To be included, articles and trials had to meet the following criteria: (1) original clinical research; (2) due to the different definitions of rare diseases in different countries and regions, such as rare diseases defined as no more than one in 2000 individuals in the European Union and no more than approximately one in 1250 in the USA [13]. We also referred to the List of Rare Diseases (https://rarediseases.org/rare-diseases). Finally, combining population bases and different definitions of rare diseases, we chose China’s First List of Rare Diseases as a reference [14]; (3) placebo conducted as the control group in the trial.

### Data extraction

YXL, CYR, and XYS authors reviewed the titles and abstracts of the retrieved articles after removing duplicates according to prespecified screening criteria. Articles that did not meet the inclusion criteria were removed. The full text of the remaining articles was independently screened by two authors (YXL and XBZ). Any discrepancies between the primary screening and full-text screening were discussed to resolve. The third (CZ) author was consulted when necessary.

Predefined data extraction tables were used to collect information for this study, two authors (YXL and XBZ) extracted the data from each trial record independently. The form of data extraction was composed of four parts: (1) General characteristics (title, author, country, year of publication, trial design, type of comparison, allocation ratio, type of disease, HM-intervention, ethics approval, informed consent, outcomes, calculation of sample size, randomization procedure, implementation of the intervention, measurement of outcome measures, and reporting of results and funding); (2) Placebo use characteristics (dosage form, administration route, the sample size of the placebo group, administration time of placebos, and controlled treatment method); (3) Reporting (TIDieR-placebo checklist [15], physically identical, quality control of placebo, assessment of safety, pharmacological inert test) and compositional characteristics of HM-placebo; (4) Efficacy and safety indicators (outcome measures and the occurrence of adverse events).

### Data analysis

Trials characteristics are presented as mean ± standard deviation (SD) for continuous variables as all were confirmed to be normally distributed, or median and interquartile range for data that were not normally distributed. The Shapiro–Wilk test was used to inspect the normality of the quantitative data. Categorical variables were reported as counts (n) and percentages (%). All data were collected and recorded in Microsoft Office Excel (Version 365). Analyses were conducted using IBM SPSS version 26 (IBM, Armonk, NY, USA).

For the assessment of safety and efficacy, we calculated the frequency of any significant difference in results between the experimental and control groups and the frequency of adverse events in each group. Among the outcome measures of the trial, the proportions of outcome measures that had a positive effect with a statistically significant difference in the intervention group compared with the control group were calculated. The primary and secondary outcomes have equal status. For the comparison of safety, we used a similar method. An intervention was considered to be safer than placebos if adverse events occurred more frequently in the placebo group.

For the reported placebo analysis, a total of 17 reporting items were counted. The first is TIDieR-Placebo which is a user-friendly guide for reporting placebo and sham control interventions that include 13 items. In addition, considering the characteristics of HM-placebo, we added 4 additional items (physically identical, quality control of placebo, assessment of safety, and pharmacological inert test) for analysis. As the five trials had no published trial protocol or publication of results, data were missing at the time of TIDieR-placebo statistics, therefore, these five studies were excluded.

To analyze associations between the number of reporting items and the above factors, Chi-squared tests, and Binary multivariable logistic regression were used. The strength of the association with each categorical characteristic was described using an odds ratio and 95% Wald confidence intervals. Funding was dichotomized as non-business versus business involvement or not reporting. HM intervention was dichotomized as single herbal versus HM formula. Ethics approval was dichotomized as reported versus not reported. Inform consent was dichotomized as reported versus not reported. The sample size was dichotomized as 1–84 versus 84–150 according to the median value. Administration time of placebos was dichotomized as ≤ 1 month versus > 1 month according to the median value. The controlled treatment method was dichotomized as placebo added to other treatments versus only placebo. Whether the control group was treated with a placebo alone, we relied on the statements in their publications. For the sensitivity analysis, we define reporting items > 7, > 8, and > 9 as relatively high-quality reporting. The seven factors we selected were based on the number of each factor after classification.

We also rated the risk of bias of the RCTs using the revised Cochrane risk of bias, version 2 (RoB 2) tool according to journal articles, study protocols, clinical trials registries information and so on.

## Results

**Fig. 1:**
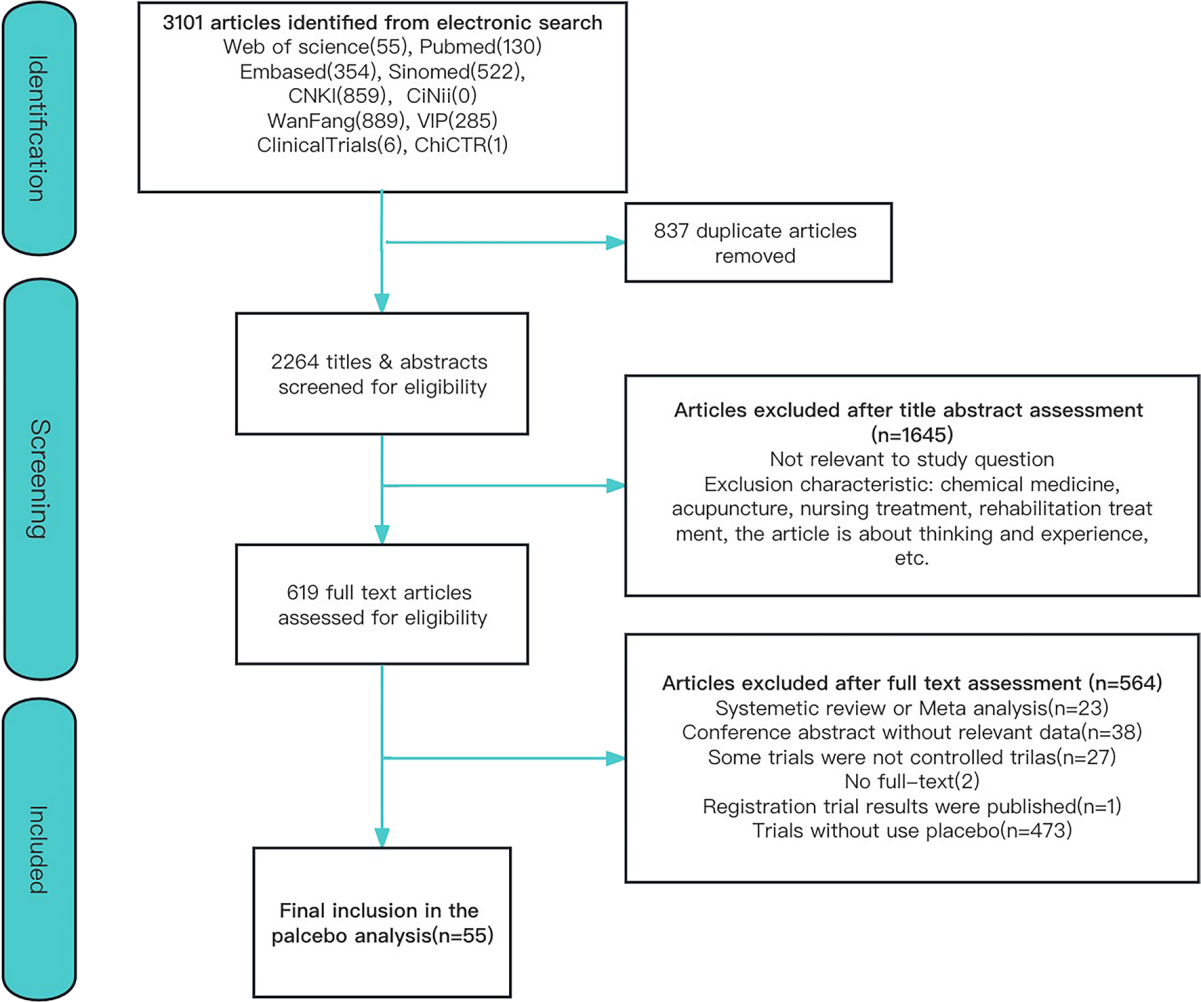
Study flow diagram

**Fig. 2:**
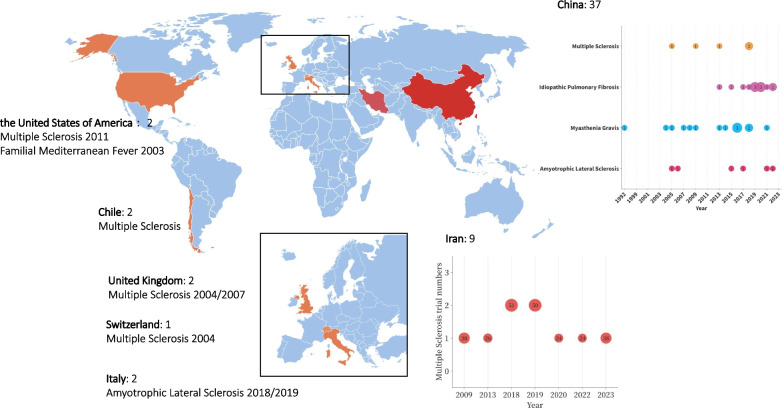
Distribution of included studies by countries’ disease and year. Data from 55 studies are included in this figure. Because of the relatively large number of trials in China and Iran, bubble plots were used for presentation. For trials in the ‘recruiting’ status, we included the time that the trial started. For trials in the ‘complete’ status, we included the time that the trial was completed. In China, the size of a scatter plot is the trial’s numbers. In Iran, the size of a scatter plot is the subject number because the disease was exclusively multiple sclerosis

A total of 3101 records were retrieved. 837 records remained after duplicates were removed. After titles and abstracts were screened, 1645 records were excluded due to irrelevant study questions, non-herbal treatment and nonclinical trials. After the examination of 619 studies, a total of 55 studies accounting for 2233 enrolled patients were included (Fig. 1). The top four diseases were multiple sclerosis (32.7%), generalized myasthenia gravis (25.5%), idiopathic pulmonary fibrosis (23.6%), and amyotrophic lateral sclerosis (12.7%). In addition, 37 rare disease studies were conducted in China, and 18 were conducted in other countries, including Iran, Chile, the United Kingdom, Italy, and the United States (Fig. 2).

### General characteristics of included trials and HM-placebo use in trials.

For the characteristics of the included trials, 2 trials [16, 17] with the largest sample size included 123 subjects, and the intervention in one trial [18] was used for up to 2 years. Four [19–22] of the included trials had crossover designs and one trial [23] had an N-of-1 design. One [24] trial used a noninferiority trial and a 1:1:1 allocation ratio. One [25] trial had a 2:1 allocation ratio for the intervention versus the control group. The HM interventions were classified as either HM formulas (42; 76.4%) or single herbs (13; 23.6%). Of 55 included trials, 27 (59.1%) did not provide their ethical approvals; 11 (20%) also did not report informed consent, and only one trial had details of informed consent. Most (53; 96.4%) of the trials chose the oral route, and the common dosage forms were granules (24; 43.6%) and tablets (8; 14.5%). In these 55 trials, the median of placebo administration time is 84 days (IQR 42–180), and the median of placebo sample size is 30 (IQR 24–54). The sample size and duration of the placebo control group were mainly distributed from 1 to 30 (29; 52.7%, 31/56) and ≤ 3 months (28; 50.9%), respectively (Table 1). Additional details about the trial design are provided in Additional file 2: Table S1.

**Table 1:** General characteristics of included trials and HM-placebo use in trials

Category	Descriptive characteristics	N = 55 (%)
*Characteristics of the trial design*		
Diseases	Multiple sclerosis	18 (32.7)
	Generalized myasthenia gravis	14 (25.5)
	Idiopathic pulmonary fibrosis	13 (23.6)
	Amyotrophic lateral sclerosis	7 (12.7)
	Systemic sclerosis	2 (3.6)
	Familial Mediterranean fever	1 (1.8)
Trials design	N-of-1	1 (1.8)
	Crossover	4 (7.3)
	Parallel placebo control	50 (90.9)
Type of comparison	non-inferiority	1 (1.8)
	superiority	49 (89.1)
	Not reported	5 (9.2)
Allocation ratio	1:1:1	1 (1.8)
	2:1	1 (1.8)
	1:1	53 (96.4)
HM intervention	Single herbal	13 (23.6)
	HM formula	42 (76.4)
Ethics approval	Yes	28 (50.9)
	Not reported	27 (49.1)
Inform consent	Yes	44 (80.0)
	Not reported	11 (20.0)
*Characteristics of placebo use*		
Dosage form	Granule	24 (43.6)
	Capsule	8 (14.5)
	Decoction	2 (3.6)
	Tablet	8 (14.5)
	Injection	2 (3.6)
	Oral-liquid	5 (9.1)
	Spray	3 (5.5)
	Plaster	1 (1.8)
	Bath	1 (1.8)
	Not reported	1 (1.8)
Administration route	Oral	53 (96.4)
	External^a^	2 (3.6)
Sample size of placebo group^b^	1—30	29 (52.7)
	31—60	15 (27.3)
	61—90	8 (14.5)
	91—120	1 (1.8)
	121—150	2 (3.6)
Duration of placebo^c^	≤ One month	10 (18.2)
	≤ Three months	28 (50.9)
	≤ Six months	8 (14.5)
	≤ One year	6 (10.9)
	> One year	3 (5.5)
Controlled treatment methods (only placebo or add-on)	Only placebo	32 (58.2)
	Add-on therapy	23 (41.8)

^a^This category refers to the HM for external use or application, such as injection, plaster, and bath

^b^Some trials had not yet completedrecruitment and were analyzed as estimated numbers

^c^We convert 'weeks' into 'months' in terms of a month with 30 day

### Reporting and compositional characteristics of HM-placebo

**Fig. 3:**
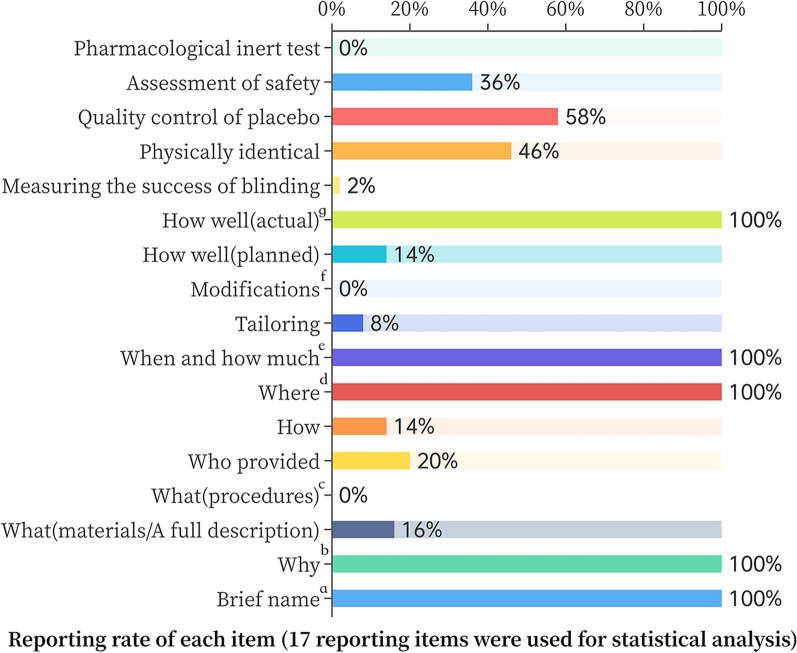
Reporting rate of each item.17 reporting items were used for statistical analysis. The 13 items from the bottom up are the TIDieR-placebo checklist. **a (Brief name)** We counted all trials that provided the name of the placebo used by the control group, including the name “placebo”. **b (Why)** We accounted for all statements of reasons for the use of placebos, which reached 100% as stated in the purpose of the trial. **c (What)** We refer to the official example of TIDieR, and the studies we included did not have eligible procedures. **d (Where)** We analyzed the data by country or hospital. **e (When and how much)** For this item, we counted drug doses for each trial. **f (Modification)** If the placebo was modified during the course of the study, describe the changes. **g (How well, actual)** If adherence or fidelity was assessed, describe the true situation. 8 (16%) trials [6, 18, 29–34] did complete reporting for the composition of the placebo. 10 (20%) trials reported that who provided placebos. 7 (14%) trials [16, 28, 31, 35–38] described the modes of delivery. 7 (14%) trials [18, 28, 30, 33, 39–41] described strategies to maintain or improve adherence. 23 (46%) trials showed that the placebo was physically identical to experimental HM, but one trial [42] the placebo was checked by a pharmacist to make sure the placebo was physically identical to experimental HM. Moreover, a trial [6] that used the same coating for the drugs, which can effectively avoid the failure of trials caused by dissimilarity. The safety assessment includes safety testing of raw materials and health testing of subjects. Only 3 (5.5%) trials [27, 30, 31] have controlled placebo raw materials, and they include microbial limits and certificates of quality. The commonly used safety tests of subjects include routine blood, urine, stool, liver function, renal function, blood test, ECG, and adverse reactions. None of the trials performed a pharmacological inert test of the placebo, even though some of the placebos used the experimental HM. More detailed information is shown in Additional file 2: Table S1. We have also translated the abstracts of the studies published in Chinese and can be found in Additional file 3

More than half of the items (11/17) were reported as relatively poor (Fig. 3). Performance was particularly poor in five areas: procedures, tailoring, modification, pharmacological inert test and measuring the success of blinding. None of the trials (0%) described procedures, activities, or processes for placebo use. 4 (8%) trials [21, 26–28] reported the placebo was personalized adapted. No one trial (0%) reported placebo was modified during the course of the study. No trials conducted pharmacological inert test. Successful blinding of the placebo design was assessed in only 1 (2%) trial [18].

For 32 trials that reported placebo compositions, 10 (18.2%; 10/55) trials included HM ingredients, and 22 (40%; 22/55) trials excluded HM ingredients in their placebos. The lower doses of the experimental HM were used primarily as placebo-containing herbal ingredients. 5% (n = 3) [35, 42, 43] and 10% (n = 4) [27, 36, 44, 45] were the most commonly used dosage percentages of HM ingredients. The placebos were mainly composed of excipients, such as soybean powder, starch, dextrin, maltodextrin, b-cyclodextrin, lactose powder, bitter taste agent, food color, etc. (Table 2). One trial [29] did not use the tested HM but also did not use additives. They used divine comedy, malt, Poria, Atractylodes, and coix seed, which are theoretically ineffective in MS.

**Table 2:** Compositional characteristics of HM-placebo

Composition of placebo	N = 32 (%)	Example
With all ingredients of tested formula	7 (21.8)	The placebo used in this study will contain 5% of the same components of JHG
With some of the ingredients of the tested formula	3 (9.4)	The placebo was simple syrup and 0.71 ml grape syrup and 0.1% St John’s wort essential oil to reach a similar color and smell to the drug
Without HM ingredients	22 (68.8)	Placebo Tablets: ImmunoGuard^®^ Clinical B. containing lactose 170 mg, calcium hydrophosphate, potato starch, microcristalline cellulose, magnesium stearate, silicagel
Drug concentrations of all HM used	N = 7^a^ (%)	Example
5%	3 (42.9)	Placebo contained 5% of the active ingredient
10%	4 (57.1)	The placebo was 10% concentration FuzhengTongluofang(shengdi, shanzhurou, nvzhenzii, huainiuxi, tusizi, wuweizi, gejie, juluo, sigualuo, sanqi) granules
Excipients	N = 51^b^ (%)	Example
Coloring agents	16 (31.4)	amaranth red, carbon black pigment, food color, caramel color
Flavoring agents	14 (27.5)	bitter taste agent, peppermint, grape syrup
Shaping agents	20 (39.2)	soybean powder, starch, and dextrin, maltodextrin, b-cyclodextrin, lactose powder, distilled water
Other agents	1 (2.0)	calcium hydrophosphate, microcristalline cellulose, magnesium stearate, silicagel, etc

^a^There were 7 trials that placebos used all tested HM

^b^Any additives of the three classes of agents used will accumulate. For example, a placebo that used both bitterness and starch and lactose was recorded as flavoring and plasticizing agents

### Efficacy and safety profiles of herbal medicine compared to placebo

Based on the results showed that among the 50 trials with the outcome indicators 23 (46%) trials did not reflect the advantage of the intervention compared with the control group. Of the 29 studies with available data on adverse events, 14 (48.3%) had 'no adverse events were observed', and 5 (17.2%) [24, 27, 30, 46, 47] studies did not show a better safety profile in the placebo group. Trials that not fitted into the extraction format were narratively summarized in Additional file 2: Table S1.

### Factors that might affect the quality of placebo reporting

The analytic factors included funding, HM-intervention, ethical approval, informed consent, sample size, administration time, and controlled treatment methods. Through the results of the Chi-squared analysis, we found that under the context that the number of report entries is delimited by 9, there was a statistically significant difference between the two groups in the rate of relatively high-quality reports of the administration time (*p* = 0.047, OR 0.1 95% CI 0.01 to 0.9) (Table 3). The results of the remaining Chi-square tests are shown in Additional file 4: Tables S2 and S3. Through the Binary multivariable logistic regression analysis of the results found, we found that the seven factors included had no statistically significant impact on the quality of the report. The results are attached in Additional file 5: Tables S4, S5 and S6. These phenomena suggest that the relatively poor design and reporting of placebos is a common problem in the rare disease field.

**Table 3:** Characteristics associated with relative-high quality reporting (items > 9)

	Reporting items > 9 (N = 7)	Reporting items ≤ 9 (N = 43)	Odds ratio (95% CI)	*P* value
HM-intervention^a^				*P* = 0.86
Single herbal	1 (8.3%)	11 (91.7%)	0.49 (0.052–4.49)	
HM-formula	6 (15.8%)	32 (84.2%)	Reference	
Ethics approval^b^				*P* = 0.82
Yes	4 (17.4%)	19 (82.6%)	1.68 (0.34–8.46)	
Not reported	3 (11.1%)	24 (88.9%)	Reference	
Inform consent^c^				*P* = 0.97
Yes	6 (15.4%)	33 (84.6%)	1.82 (0.20–16.9)	
Not reported	1 (9.1%)	10 (90.9%)	Reference	
Sample size^d^				*P* = 1.00
1–84	4 (13.8%)	25 (86.2%)	0.96 (0.19–4.83)	
84–150	3 (14.3%)	18 (85.7%)	Reference	
Administration time^e^				*P* = 0.047
≤ 1 month	1 (3.6%)	27 (96.4%)	0.1 (0.01–0.90)	
> 1 month	6 (27.3%)	16 (72.7%)	Reference	
Funding^f^				*P* = 0.91
Non-business	4 (16.7%)	20 (83.3%)	1.53 (0.31–7.69)	
Business or not reporting	3 (11.5%)	23 (88.5%)	Reference	
Therapy^g^				*P* = 0.72
Add-on	5 (17.2%)	24 (82.8%)	1.98 (0.53–11.35)	
Only placebo	2 (9.5%)	19 (90.5%)	Reference	

^a^HM-intervention was dichotomized as singer herbal versus HM formula

^b^Ethics approval was dichotomized as reported versus not reported

^c^Inform consent was dichotomized as reported versus not reported

^d^The Sample size was dichotomized as 1–84 versus 84–150 according to the median value

^e^Administration time of placebos was dichotomized as ≤ 1 month versus > 1 month according to the median value

^f^Funding was dichotomized as non-business versus business involvement or not reporting

^g^The controlled treatment method was dichotomized as placebo added to other treatments versus only placebo

### RoB2 assessment

The results of the risk of bias assessment were as follows. Regarding performance bias, 7 studies were evaluated high risk and 32 studies were evaluated some concerns. The missing outcome data domain is low risk. Randomization process and interventions implementation more than half existing some concerns. In terms of random sequence generation, only 14 RCTs described the specific randomization process. 9 studies without appropriate measurements of the outcome (Additional file 6: Fig. S1).

## Discussion

A total of 55 herbal clinical trials were conducted for 6 rare diseases. The overall quality of HM-placebo in the field of rare diseases was poor. In the placebo preparation phase, almost all trials whether by technical or manual methods, did not assess placebo for safety, inertia, or blinding. None of the studies reported the overall details of the placebo. Nearly half of the trials provided their ethical approvals. In terms of safety, 17.2% of the clinical trials showed that placebos did not perform better than the intervention. Meanwhile, the quality of the included RCTS also deserves our attention.

### Application of assessment methods

Superior placebo preparation is fundamental not only to trials but also to good placebo reporting. Placebo preparation of HM is limited due to its special sense of smell and taste, which is much more complicated compared to compound drugs. The materials used in the preparation of the placebo should be selected in strict accordance with the quality standards of pharmaceutical excipients and the hygienic standards for the use of food additives. Under the premise of ensuring safety and no active substances, the characteristics of various excipients and additives were compared to obtain the optimal formulation of each placebo [10]. For the raw materials of the placebo, we should ensure their safety and test items such as pesticide residue limit, microbial limit, aflatoxin, and nitrosamine. The safety of the drug during the trial is the focus of the subjects, especially the HM-placebo, which is often complex in composition, and its combination of substances may cause adverse reactions in the subjects. Therefore, safety monitoring during the trial is particularly important. The most used tests include routine blood tests, routine urine tests, electrocardiograms, liver function tests, and kidney function tests. Due to the vulnerability of patients with rare diseases, we should pay more attention to safety monitoring.

There are some difficulties in the preparation of placebos, some HM placebos are designed to contain 5% to 10% of the standard dose and to better simulate the specific odor and taste of HM extract, the remaining formulation consists of suitable excipient and additive. Therefore, the pharmacological inert test was recommended. The phenomenon can be seen from our statistical results that some trials did not show the superiority of intervention that used low doses of tested drugs over placebo, which may be related to the characteristics of rare diseases, but also cannot prove that this is not related to low doses of HM. Therefore, the detection of drug inertia may help us to support our trial results reasonably. The content of active ingredients was determined by pharmacodynamic tests, thin-layer chromatography, high-performance liquid chromatography, infrared spectroscopy, and ultraviolet spectroscopy.

One of the most important properties of placebos is similarity. The evaluation of similarity can be divided into manual evaluation and intelligent evaluation. In terms of manual evaluation, a study proposed three comparative evaluation methods, tested three dosage forms (oral liquid, capsule, granule), and compared the better evaluation method for other researchers to reference [48]. Intelligent evaluation methods include electronic noses, electronic tongues, and visual sensors [49–51]. Intelligent sensory technology can realize the objective evaluation of traditional HM-placebo. Machine vision technology, electronic nose and electronic tongue technology are used to collect relevant data for each sample, and the characteristics of placebo data are analyzed by different statistical methods. It can avoid the subjective factors caused by artificial scoring to some extent and is a powerful auxiliary for the evaluation of placebo. The similarity is fundamental to the success of blinding. According to Consolidated Standards of Reporting Trials (CONSORT), investigators are required to report not only whether blinding was performed but also the details and procedures of blinding and whether blinding was successfully performed. In 1996, James et al. systematically introduced the theory and application of James’ index through an application example of a clinical randomized controlled trial [52]. Later, Bang et al. further proposed Bang's index to evaluate blinding success [53]. The findings from RoB 2.0 indicate that a significant proportion of trials encountered challenges related to randomization and intervention delivery. Among the pivotal considerations in this evaluation is the aspect of blinding. The successful implementation of blinding crucially hinges upon the quality and similarity of placebos employed. Consequently, the role of placebo similarity is a factor of paramount importance that cannot be underestimated. The quality of placebos stands in close correlation with the conduct of clinical trials and, indeed, can be the determinant in ensuring the smooth implementation of a clinical trial. To improve the quality of HM clinical trials in the field of rare diseases and to produce higher quality clinical evidence under the premise of fully protecting the interests of subjects.

### Ethical considerations

From the results of our trial, we can also see that the ethical review required by international authoritative organizations has not been perfect, and ethical review in the field of rare diseases should be fully considered. Given the genetic characteristics of rare diseases, children account for about 50% of patients with rare diseases. Thus, researchers and clinicians should always keep in mind that additional ethical considerations exist [54]. First, the researcher must provide adequate informed consent. How to get them to fully understand placebos is something researchers must carefully consider. Full informed consent should not simply be stated as ‘signed’. In the cases of pediatric patients, vulnerable patients are unable to provide consent, and trial details also need to be provided to their legal guardians. The summary of both the laboratory and clinical data accumulated to date was explained to subjects and their legal guardians in simple nontechnical language.

Placebos are a key link to trial design and ethics, and we also briefly analyze the ethical considerations for patients with rare diseases in different contexts. First, alternative trial designs, such as crossover studies, are needed. Crossover studies that include a placebo treatment period face the same ethical challenges as placebo-controlled studies using monotherapy [55]. Second, in noninferiority studies, the null hypothesis of inferiority must be rejected, demonstrating that experimental treatment is not inferior to the active comparator arm. If an established effective therapy (EET) is superior to placebos in prior clinical trials, performance on that EET should be able to be used as a control [56]. However, patient populations recruited for studies at different times may not be the same, and EET effects over time and in different study populations may differ [57]. Thus, comparability between the results of past placebo-controlled trials and the performance of the active control in the new trial cannot be demonstrated. The non-inferiority study design is inherently marked by its safety features. However, its application in the context of rare diseases poses a significant challenge, primarily because many rare diseases lack established, standard, and effective treatment modalities. Third, clinical trials in which an experimental agent or its placebo control is added to an EET are feasible and ethically acceptable [58]. However, this situation may affect the accurate assessment of the efficacy and safety of the tested drug. For rare diseases and smaller populations, researchers have always carried out studies on trial design to conduct clinical trials reasonably and effectively in the future [59–62]. We believe these considerations are consistent with both the need to develop improved therapies and the legitimate concerns for individual patient protection.

### Reporting of placebo

There is no standard checklist for the quality evaluation of HM-placebo. Therefore, we refer to the TIDieR-placebo report for statistics and analysis in this study. However, we found that there was a need to develop a reporting checklist for HM-placebos. Based on our results, we recommend the following additional reporting information. First, the source of the raw materials should be reported. Regardless of the dosage form used, different sources of materials and different production personnel may affect the quality of the final placebo. Therefore, the entire control method for placebos should be reported. Second, the method of similarity assessment should be reported. Most of the included studies briefly describe the similarity in words. We recommend reporting on how to ensure their placebo is similar to HM. Researchers could choose one or more reasonable similarity evaluation indices mentioned above. As a special preparation, the dosage of each component of the HM placebo should be clearly defined. More attention should be paid to materials with pharmacological effects. For the addition of HM, regardless of whether it is a trial drug, we recommend a pharmacological inert test or a pharmacodynamic test to ensure that it has no pharmacological effect. At the same time, there is no optimal imitation method to explain why tested HM is added to placebos.

## Strengths and limitations

This study has some limitations that must be considered. First, the localization study used only 10 databases, potentially limiting the number of studies included. Second, search terms are extensive, simple, and free. At present, there is no unified definition of rare diseases. Searches were limited to titles and abstracts, so it is possible that some articles were excluded from the study if the search term was not mentioned in the title or abstract. Third, this study focused on describing the use and reporting quality of HM-placebo, the logistic regression analysis was helpful to describe the reporting of HM-placebo in more depth. However, the limited trials included in this study may pose a challenge to the modeling process. To the best of our knowledge, this is the first study that summarized the use of HM-placebos in rare diseases. As our predefined selection criteria were wide, there are differences in the reporting content of each trial, but this can better reflect the problems with the current trial reporting. We did not have a priori restrictions regarding language, but our search only identified full-length articles in English and Chinese. We have done our best to retrieve all available data and to include factors that might have influenced the quality of placebo reporting, we believe that the general trends indicated by the analyses we have made are valid even if the information we have included in the analyses is incomplete .

## Conclusion

Herbal medicines encompass the combination of practices of indigenous systems of medicine and several therapeutic experiences of many previous generations. Which delivers valuable references to the selection, preparation, and application of herbal formulation for the treatment, control, and management of rare diseases. Currently, certain countries have established well-defined herbal diagnostic and treatment protocols within the domains of hepatolenticular degeneration, multiple sclerosis, retinitis pigmentosa and so on. These endeavors have been instrumental in the ongoing exploration of the therapeutic potential of herbal medicine, offering promising pharmaceutical alternatives for orphan diseases. The ongoing progression of clinical trials in this sphere simultaneously serves as a cornerstone for future drug development. The use of HM-placebo in the field of rare diseases has increased in recent years. Despite its advantages in the evaluation of herbal products, its use in rare diseases is still relatively poor. As guidelines developed specifically from the point of view of placebos have increased over the years, they have matured and are used in controlled trials. However, in the future, more emphasis should be placed on a systematic reporting checklist of the compositions of HM-placebo to obtain quality publications. Regardless of the missing information in earlier reporting, while HM-placebo has been implemented in research, the use of HM-placebo in rare disease trials is highly recommended, and researchers are encouraged to apply it.

### Supplementary Information

**Additional file 1.** Retrieval strategies.

**Additional file 2.** Summary of trials information.

**Additional file 3.** Summary of abstracts of studies published in Chinese.

**Additional file 4.** Chi-squared analysis.

**Additional file 5.** Logistic regression.

**Additional file 6.** Risk of bias of included studies.

## Data Availability

Not applicable. Placebo-related data were collected from clinical trials of HM in the field of rare diseases and were not derived from a single data set.

## Abbreviations

ChiCTR: Chinese Clinical Trials Registry
CiNii: National Institute of Informatics Support Academic Information Services
CNKI: China National Knowledge Infrastructure
CONSORT: Consolidated Standards of Reporting Trials
ECG: Electrocardiogram
EET: Established effective therapy
HM: Herbal medicine
IQR: Inter quartile range
MS: Multiple sclerosis
N-of-1: Randomized controlled trial in individual patient
OR: Odds ratio
RCT: Randomized controlled trial
SD: Standard deviation
TIDieR: Template for intervention description and replication
95% CI: 95% confidence interval
