# Supplementary material for: Qualified placebo for trials of herbal medicine treatment in rare diseases? A cross-sectional analysis
Source: Orphanet J Rare Dis. 2023 Nov 30;18:373. doi: 10.1186/s13023-023-02987-w (PMC10691121; doi:10.1186/s13023-023-02987-w)
Supplement: Supplementary file 1 — Additional file 1. Retrieval strategies. [file 13023_2023_2987_MOESM1_ESM.docx]

Supplementary Material 1

(Search strategies)

**Qualified Placebo for Trials of Herbal Medicine Treatment in Rare Diseases? A Cross-Sectional Analysis**

**Yixuan Li, Peipei Du Xuebin Zhang, Chenyu Ren, XinYi Shi, XingLu Dong^*^, Chi Zhang^*^**

*** Correspondence:**

Chi Zhang

[saga618@126.com](mailto:saga618@126.com)

Xinglu Dong

[arthasdxl@163.com](mailto:arthasdxl@163.com)

1. **Pubmed:**

(((((((((((((((((((((((((21-Hydroxylase Deficiency[MeSH Terms]) OR (Albinism[MeSH Terms])) OR (Alport Syndrome[MeSH Terms])) OR (Amyotrophic Lateral Sclerosis[MeSH Terms])) OR (Angelman Syndrome[MeSH Terms])) OR (Arginase Deficiency[MeSH Terms])) OR (Asphyxiating Thoracic Dystrophy[MeSH Terms])) OR (Atypical Hemolytic Uremic Syndrome[MeSH Terms])) OR (Autoimmune Encephalitis[MeSH Terms])) OR (Autoimmune Hypophysitis[MeSH Terms])) OR (Autoimmune Insulin Receptopathy[MeSH Terms])) OR (Beta-ketothiolase Deficiency[MeSH Terms])) OR (Biotinidase Deficiency[MeSH Terms])) OR (Cardic Ion Channelopathies[MeSH Terms])) OR (Carnitine Deficiency[MeSH Terms])) OR (Castleman Disease[MeSH Terms])) OR (Charcot-Marie-Tooth Disease[MeSH Terms])) OR (Citrullinemia[MeSH Terms])) OR (Congenital Adrenal Hypoplasia[MeSH Terms])) OR (Congenital Hyperinsulinemic Hypoglycemia[MeSH Terms])) OR (Congenital Myasthenic Syndrome[MeSH Terms])) OR (Congenital Myotonia Syndrome[MeSH Terms])) OR (Congenital Scoliosis[MeSH Terms])) OR (Coronary Artery Ectasia[MeSH Terms])) AND ((((((((((( "Drugs, Chinese Herbal/administration and dosage"[Mesh] OR "Drugs, Chinese Herbal/adverse effects"[Mesh] OR "Drugs, Chinese Herbal/therapeutic use"[Mesh] )) OR (Chinese Drugs, Plant)) OR (Chinese Herbal Drugs)) OR (Herbal Drugs, Chinese)) OR (Plant Extracts, Chinese)) OR (Chinese Plant Extracts)) OR (Extracts, Chinese Plant))) OR ((((((("Medicine, Korean Traditional"[Mesh]) OR (Traditional Medicine, Korean)) OR (Traditional Medicine, Korea)) OR (Korea Traditional Medicine)) OR (Medicine, Korea Traditional)) OR (Sasang Constitutional Medicine)) OR (Medicine, Sasang Constitutional))) OR (((("Medicine, Kampo"[Majr]) OR (Kanpo Medicine)) OR (Medicine, Kanpo)) OR (Kampo Medicine)))) AND (((randomized controlled trials) OR (clinical trials)))

Results:18

(((((((((((((Diamond-Blackfan Anemia[MeSH Terms])) OR (Fabry Disease[MeSH Terms])) OR (Erdheim-Chester Disease[MeSH Terms])) OR (Familial Mediterranean Fever[MeSH Terms])) OR (Fanconi Anemia[MeSH Terms])) OR (Galactosemia[MeSH Terms])) OR (Gaucher’s Disease[MeSH Terms])) OR (Generalized Myasthenia Gravis[MeSH Terms])) OR (Gitelman Syndrome[MeSH Terms])) OR (Glutaric Acidemia Type I[MeSH Terms])) OR (Glutaric Acidemia Type I[MeSH Terms])) AND ((((((((((( "Drugs, Chinese Herbal/administration and dosage"[Mesh] OR "Drugs, Chinese Herbal/adverse effects"[Mesh] OR "Drugs, Chinese Herbal/therapeutic use"[Mesh] )) OR (Chinese Drugs, Plant)) OR (Chinese Herbal Drugs)) OR (Herbal Drugs, Chinese)) OR (Plant Extracts, Chinese)) OR (Chinese Plant Extracts)) OR (Extracts, Chinese Plant))) OR ((((((("Medicine, Korean Traditional"[Mesh]) OR (Traditional Medicine, Korean)) OR (Traditional Medicine, Korea)) OR (Korea Traditional Medicine)) OR (Medicine, Korea Traditional)) OR (Sasang Constitutional Medicine)) OR (Medicine, Sasang Constitutional))) OR (((("Medicine, Kampo"[Majr]) OR (Kanpo Medicine)) OR (Medicine, Kanpo)) OR (Kampo Medicine)))) AND (((randomized controlled trials) OR (clinical trials))) results:10

((((((((((((((((((Hemophilia[MeSH Terms]) OR (Hepatolenticular Degeneration[MeSH Terms])) OR (Wilson Disease[MeSH Terms])) OR (Hereditary Angioedema[MeSH Terms])) OR (Hereditary Epidermolysis Bullosa[MeSH Terms])) OR (Hereditary Fructose Intolerance[MeSH Terms])) OR (Hereditary Hypomagnesemia[MeSH Terms])) OR (Hereditary Multi-infarct Dementia[MeSH Terms])) OR (Hereditary Spastic Paraplegia[MeSH Terms])) OR (Holocarboxylase Synthetase Deficiency[MeSH Terms])) OR (Homocysteinemia[MeSH Terms])) OR (Homozygous Hypercholesterolemia[MeSH Terms])) OR (Huntington Disease[MeSH Terms])) OR (Hyperornithinaemia-Hyperammonaemia-Homocitrullinuria Syndrome[MeSH Terms])) OR (Hyperphenylalaninemia[MeSH Terms])) OR (Hypophosphatasia[MeSH Terms])) OR (Hypophosphatemic Rickets[MeSH Terms])) AND ((((((((((( "Drugs, Chinese Herbal/administration and dosage"[Mesh] OR "Drugs, Chinese Herbal/adverse effects"[Mesh] OR "Drugs, Chinese Herbal/therapeutic use"[Mesh] )) OR (Chinese Drugs, Plant)) OR (Chinese Herbal Drugs)) OR (Herbal Drugs, Chinese)) OR (Plant Extracts, Chinese)) OR (Chinese Plant Extracts)) OR (Extracts, Chinese Plant))) OR ((((((("Medicine, Korean Traditional"[Mesh]) OR (Traditional Medicine, Korean)) OR (Traditional Medicine, Korea)) OR (Korea Traditional Medicine)) OR (Medicine, Korea Traditional)) OR (Sasang Constitutional Medicine)) OR (Medicine, Sasang Constitutional))) OR (((("Medicine, Kampo"[Majr]) OR (Kanpo Medicine)) OR (Medicine, Kanpo)) OR (Kampo Medicine)))) AND (((randomized controlled trials) OR (clinical trials))) results:8

((((((((((((((((Idiopathic Cardiomyopathy[MeSH Terms]) OR (Idiopathic Hypogonadotropic Hypogonadism[MeSH Terms])) OR (Idiopathic Pulmonary Arterial Hypertension[MeSH Terms])) OR (Idiopathic Pulmonary Fibrosis[MeSH Terms])) OR (IgG4 related Disease[MeSH Terms])) OR (Inborn Errors of Bile Acid Synthesis[MeSH Terms])) OR (Isovaleric Acidemia[MeSH Terms])) OR (Kallmann Syndrome[MeSH Terms])) OR (Langerhans Cell Histiocytosis[MeSH Terms])) OR (Langerhans Cell Histiocytosis[MeSH Terms])) OR (Leber Hereditary Optic Neuropathy[MeSH Terms])) OR (Long Chain 3-hydroxyacyl-CoA Dehydrogenase Deficiency[MeSH Terms])) OR (Lymphangioleiomyomatosis[MeSH Terms])) OR (Lysinuric Protein Intolerance[MeSH Terms])) OR (Lysosomal Acid Lipase Deficiency[MeSH Terms])) AND ((((((((((( "Drugs, Chinese Herbal/administration and dosage"[Mesh] OR "Drugs, Chinese Herbal/adverse effects"[Mesh] OR "Drugs, Chinese Herbal/therapeutic use"[Mesh] )) OR (Chinese Drugs, Plant)) OR (Chinese Herbal Drugs)) OR (Herbal Drugs, Chinese)) OR (Plant Extracts, Chinese)) OR (Chinese Plant Extracts)) OR (Extracts, Chinese Plant))) OR ((((((("Medicine, Korean Traditional"[Mesh]) OR (Traditional Medicine, Korean)) OR (Traditional Medicine, Korea)) OR (Korea Traditional Medicine)) OR (Medicine, Korea Traditional)) OR (Sasang Constitutional Medicine)) OR (Medicine, Sasang Constitutional))) OR (((("Medicine, Kampo"[Majr]) OR (Kanpo Medicine)) OR (Medicine, Kanpo)) OR (Kampo Medicine)))) AND (((randomized controlled trials) OR (clinical trials))) results:65

(((((((((((((Maple Syrup Urine Disease[MeSH Terms]) OR (Marfan Syndrome[MeSH Terms])) OR (McCune-Albright Syndrome[MeSH Terms])) OR (Medium Chain Acyl-CoA Dehydrogenase Deficiency[MeSH Terms])) OR (Methylmalonic Academia[MeSH Terms])) OR (Mitochodrial Encephalomyopathy[MeSH Terms])) OR (Mucopolysaccharidosis[MeSH Terms])) OR (Multifocal Motor Neuropathy[MeSH Terms])) OR (Multiple Acyl-CoA Dehydrogenase Deficiency[MeSH Terms])) OR (Multiple Sclerosis[MeSH Terms])) OR (Multiple System Atrophy[MeSH Terms])) OR (Myotonic Dystrophy[MeSH Terms])) AND ((((((((((( "Drugs, Chinese Herbal/administration and dosage"[Mesh] OR "Drugs, Chinese Herbal/adverse effects"[Mesh] OR "Drugs, Chinese Herbal/therapeutic use"[Mesh] )) OR (Chinese Drugs, Plant)) OR (Chinese Herbal Drugs)) OR (Herbal Drugs, Chinese)) OR (Plant Extracts, Chinese)) OR (Chinese Plant Extracts)) OR (Extracts, Chinese Plant))) OR ((((((("Medicine, Korean Traditional"[Mesh]) OR (Traditional Medicine, Korean)) OR (Traditional Medicine, Korea)) OR (Korea Traditional Medicine)) OR (Medicine, Korea Traditional)) OR (Sasang Constitutional Medicine)) OR (Medicine, Sasang Constitutional))) OR (((("Medicine, Kampo"[Majr]) OR (Kanpo Medicine)) OR (Medicine, Kanpo)) OR (Kampo Medicine)))) AND (((randomized controlled trials) OR (clinical trials)))

Results:6

(((((((((((((((((N-acetylglutamate Synthase Deficiency[MeSH Terms]) OR (Neonatal Diabetes Mellitus[MeSH Terms])) OR (Neuromyelitis Optica[MeSH Terms])) OR (Niemann-Pick Disease[MeSH Terms])) OR (Non-Syndromic Deafness[MeSH Terms])) OR (Noonan Syndrome[MeSH Terms])) OR (Ornithine Transcarbamylase Deficiency[MeSH Terms])) OR (Osteogenesis Imperfecta[MeSH Terms])) OR (Brittle Bone Disease[MeSH Terms])) OR (Parkinson Disease[MeSH Terms])) OR (Paroxysmal Nocturnal Hemoglobinuria[MeSH Terms])) OR (Peutz-Jeghers Syndrome[MeSH Terms])) OR (Phenylketonuria[MeSH Terms])) OR (POEMS Syndrome[MeSH Terms])) OR (Porphyria[MeSH Terms])) OR (Prader-Willi Syndrome[MeSH Terms])) AND ((((((((((( "Drugs, Chinese Herbal/administration and dosage"[Mesh] OR "Drugs, Chinese Herbal/adverse effects"[Mesh] OR "Drugs, Chinese Herbal/therapeutic use"[Mesh] )) OR (Chinese Drugs, Plant)) OR (Chinese Herbal Drugs)) OR (Herbal Drugs, Chinese)) OR (Plant Extracts, Chinese)) OR (Chinese Plant Extracts)) OR (Extracts, Chinese Plant))) OR ((((((("Medicine, Korean Traditional"[Mesh]) OR (Traditional Medicine, Korean)) OR (Traditional Medicine, Korea)) OR (Korea Traditional Medicine)) OR (Medicine, Korea Traditional)) OR (Sasang Constitutional Medicine)) OR (Medicine, Sasang Constitutional))) OR (((("Medicine, Kampo"[Majr]) OR (Kanpo Medicine)) OR (Medicine, Kanpo)) OR (Kampo Medicine)))) AND (((randomized controlled trials) OR (clinical trials)))

Results:36

((((((((((((((((((((Primary Combined Immune Deficiency[MeSH Terms]) OR (Primary Hereditary Dystonia[MeSH Terms])) OR (Primary Light Chain Amyloidosis[MeSH Terms])) OR (Progressive Familial Intrahepatic Cholestasis[MeSH Terms])) OR (Progressive Muscular Dystrophy[MeSH Terms])) OR (Propionic Acidemia[MeSH Terms])) OR (Pulmonary Alveolar Proteinosis[MeSH Terms])) OR (Pulmonary Cystic Fibrosis[MeSH Terms])) OR (Retinitis Pigmentosa[MeSH Terms])) OR (Retinoblastoma[MeSH Terms])) OR (Severe Congenital Neutropenia[MeSH Terms])) OR (Severe Myoclonic Epilepsy in Infancy[MeSH Terms])) OR (Sickle Cell Disease[MeSH Terms])) OR (Silver-Russell Syndrome[MeSH Terms])) OR (Sitosterolemia[MeSH Terms])) OR (Spinal and Bulbar Muscular Atrophy[MeSH Terms])) OR (Kennedy Disease[MeSH Terms])) OR (Spinal Muscular Atrophy[MeSH Terms])) OR (Spinocerebellar Ataxia[MeSH Terms])) AND ((((((((((( "Drugs, Chinese Herbal/administration and dosage"[Mesh] OR "Drugs, Chinese Herbal/adverse effects"[Mesh] OR "Drugs, Chinese Herbal/therapeutic use"[Mesh] )) OR (Chinese Drugs, Plant)) OR (Chinese Herbal Drugs)) OR (Herbal Drugs, Chinese)) OR (Plant Extracts, Chinese)) OR (Chinese Plant Extracts)) OR (Extracts, Chinese Plant))) OR ((((((("Medicine, Korean Traditional"[Mesh]) OR (Traditional Medicine, Korean)) OR (Traditional Medicine, Korea)) OR (Korea Traditional Medicine)) OR (Medicine, Korea Traditional)) OR (Sasang Constitutional Medicine)) OR (Medicine, Sasang Constitutional))) OR (((("Medicine, Kampo"[Majr]) OR (Kanpo Medicine)) OR (Medicine, Kanpo)) OR (Kampo Medicine)))) AND (((randomized controlled trials) OR (clinical trials)))

Results:4

(((((((((((Systemic Sclerosis[MeSH Terms]) OR (Tetrahydrobiopterin Deficiency[MeSH Terms])) OR (Tuberous Sclerosis Complex[MeSH Terms])) OR (Tyrosinemia[MeSH Terms])) OR (Very Long Chain Acyl-CoA Dehydrogenase Deficiency[MeSH Terms])) OR (Williams Syndrome[MeSH Terms])) OR (Wiskott-Aldrich Syndrome[MeSH Terms])) OR (X-linked Agammaglobulinemia[MeSH Terms])) OR (X-linked Adrenoleukodystrophy[MeSH Terms])) OR (X-linked Lymphoproliferative Disease[MeSH Terms])) AND ((((((((((( "Drugs, Chinese Herbal/administration and dosage"[Mesh] OR "Drugs, Chinese Herbal/adverse effects"[Mesh] OR "Drugs, Chinese Herbal/therapeutic use"[Mesh] )) OR (Chinese Drugs, Plant)) OR (Chinese Herbal Drugs)) OR (Herbal Drugs, Chinese)) OR (Plant Extracts, Chinese)) OR (Chinese Plant Extracts)) OR (Extracts, Chinese Plant))) OR ((((((("Medicine, Korean Traditional"[Mesh]) OR (Traditional Medicine, Korean)) OR (Traditional Medicine, Korea)) OR (Korea Traditional Medicine)) OR (Medicine, Korea Traditional)) OR (Sasang Constitutional Medicine)) OR (Medicine, Sasang Constitutional))) OR (((("Medicine, Kampo"[Majr]) OR (Kanpo Medicine)) OR (Medicine, Kanpo)) OR (Kampo Medicine)))) AND (((randomized controlled trials) OR (clinical trials)))

Results:23

1. **Embased**

No. Query Results Results Date

#18. #13 AND #14 AND #16 246 15 Feb 2023

#17. #13 AND #14 AND #15 108 15 Feb 2023

#16. 'leber hereditary optic neuropathy' OR 'long 473,939 15 Feb 2023

chain 3-hydroxyacyl-coa' OR 'dehydrogenase

deficiency' OR 'lymphangioleiomyomatosis' OR

'lysinuric protein intolerance' OR 'lysosomal

acid lipase deficiency' OR 'maple syrup urine

disease' OR 'marfan syndrome' OR 'mccune-albright

syndrome' OR 'medium chain acyl-coa dehydrogenase

deficiency' OR 'methylmalonic academia' OR

'mitochodrial encephalomyopathy' OR

'mucopolysaccharidosi' OR 'multifocal motor

neuropathy' OR 'multiple acyl-coa dehydrogenase

deficiency' OR 'multiple sclerosis' OR 'multiple

system atrophy' OR 'myotonic dystrophy' OR

'n-acetylglutamate synthase deficiency' OR

'neonatal diabetes mellitus' OR 'neuromyelitis

optica' OR 'niemann-pick disease' OR

'non-syndromic deafness' OR 'noonan syndrome' OR

'ornithine transcarbamylase deficiency' OR

'osteogenesis imperfecta' OR 'brittle bone

disease' OR 'young-onset parkinson disease' OR

'early-onset parkinson disease' OR 'paroxysmal

nocturnal hemoglobinuria' OR 'peutz-jeghers

syndrome' OR 'phenylketonuria' OR 'poems

syndrome' OR 'porphyria' OR 'prader-willi

syndrome' OR 'primary combined immune deficiency'

OR 'primary hereditary dystonia' OR 'primary

light chain amyloidosis' OR 'progressive familial

intrahepatic cholestasis' OR 'progressive

muscular dystrophy' OR 'propionic acidemia' OR

'pulmonary alveolar proteinosis' OR 'pulmonary

cystic fibrosis' OR 'retinitis pigmentosa' OR

'retinoblastoma' OR 'severe congenital

neutropenia' OR 'severe myoclonic epilepsy in

infancy' OR 'dravet syndrome' OR 'sickle cell

disease' OR 'silver-russell syndrome' OR

'sitosterolemia' OR 'kennedy disease' OR 'spinal

muscular atrophy' OR 'spinocerebellar ataxia' OR

'systemic sclerosis' OR 'tetrahydrobiopterin

deficiency' OR 'tuberous sclerosis complex' OR

'tyrosinemia' OR 'very long chain acyl-coa

dehydrogenase deficiency' OR 'williams syndrome'

OR 'wiskott-aldrich syndrome' OR 'x-linked

agammaglobulinemia' OR 'x-linked

adrenoleukodystrophy' OR 'x-linked

lymphoproliferative disease'

#15. '21-hydroxylase deficiency' OR 'albinism' OR 417,699 15 Feb 2023

'alport syndrome' OR 'amyotrophic lateral

sclerosis' OR 'angelman syndrome' OR 'arginase

deficiency' OR 'asphyxiating thoracic dystrophy'

OR 'jeune syndrome' OR 'atypical hemolytic uremic

syndrome' OR 'autoimmune encephalitis' OR

'autoimmune hypophysitis' OR 'autoimmune insulin

receptopathy' OR 'type b insulin resistance' OR

'beta-ketothiolase deficiency' OR 'biotinidase

deficiency' OR 'cardic ion channelopathies' OR

'carnitine deficiency' OR 'castleman disease' OR

'charcot-marie-tooth disease' OR 'citrullinemia'

OR 'congenital adrenal hypoplasia' OR 'congenital

hyperinsulinemic hypoglycemia' OR 'congenital

myasthenic syndrome' OR 'congenital myotonia

syndrome' OR 'non-dystrophic myotonia' OR

'congenital scoliosis' OR 'coronary artery

ectasia' OR 'diamond-blackfan anemia' OR

'erdheim-chester disease' OR 'fabry disease' OR

'familial mediterranean fever' OR 'fanconi

anemia' OR 'galactosemia' OR 'gaucher disease' OR

'generalized myasthenia gravis' OR 'gitelman

syndrome' OR 'glutaric acidemia type i' OR

'hemophilia' OR 'hepatolenticular degeneration'

OR 'wilson disease' OR 'hereditary angioedema' OR

'hereditary' OR 'epidermolysis bullosa' OR

'hereditary fructose intolerance' OR 'hereditary

hypomagnesemia' OR 'hereditary multi-infarct

dementia' OR 'hereditary spastic paraplegia' OR

'holocarboxylase synthetase deficiency' OR

'homocysteinemia' OR 'homozygous

hypercholesterolemia' OR 'huntington disease' OR

'hyperornithinaemia-hyperammonaemia-

homocitrullinuria syndrome' OR

'hyperphenylalaninemia' OR 'hypophosphatasia' OR

'hypophosphatemic rickets' OR 'idiopathic

cardiomyopathy' OR 'idiopathic hypogonadotropic

hypogonadism' OR 'idiopathic pulmonary arterial

hypertension' OR 'idiopathic pulmonary fibrosis'

OR 'igg4 related disease' OR 'inbornerrors of

bile acid synthesis' OR 'isovaleric acidemia' OR

'kallmann syndrome' OR 'langerhans cell

histiocytosis'

#14. #10 OR #11 OR #12 1,209,899 15 Feb 2023

#13. #1 OR #2 OR #3 OR #4 OR #5 OR #6 OR #7 OR #8 OR 575,618 15 Feb 2023

#9

#12. 'double blind procedure' 204,361 15 Feb 2023

#11. 'clinical trial (topic)' 201,856 15 Feb 2023

#10. 'randomized controlled trial' 1,012,774 15 Feb 2023

#9. 'medicinal plant' 115,337 15 Feb 2023

#8. 'japanese medicine' 542 15 Feb 2023

#7. 'kampo medicine (drug)' 1,512 15 Feb 2023

#6. 'traditional medicine' 56,532 15 Feb 2023

#5. 'chinese medicine' 208,068 15 Feb 2023

#4. 'herbaceous agent' 59,540 15 Feb 2023

#3. 'plant extract' 182,203 15 Feb 2023

#2. 'alternative medicine' 74,008 15 Feb 2023

#1. 'traditional medicine'/exp OR 'traditional 149,716 15 Feb 2023

medicine'

1. **Web of science**

- WOS.SCI: 1900 to 2023

- WOS.AHCI: 1975 to 2023

- WOS.BHCI: 2005 to 2023

- WOS.BSCI: 2005 to 2023

- WOS.ESCI: 2005 to 2023

- WOS.ISTP: 1990 to 2023

- WOS.SSCI: 1900 to 2023

- WOS.ISSHP: 1990 to 2023

1: TI=(21-Hydroxylase Deficiency) OR TI=(Albinism) OR TI=(Alport Syndrome) OR TI=(Amyotrophic Lateral Sclerosis) OR TI=(Angelman Syndrome) OR TI=(Arginase Deficiency) OR TI=(Asphyxiating Thoracic Dystrophy) OR TI=(Jeune Syndrome) OR TI=(Atypical Hemolytic Uremic Syndrome) OR TI=(Autoimmune Encephalitis) OR TI=(Autoimmune Hypophysitis) OR TI=(Autoimmune Insulin Receptopathy) OR TI=(Type B insulin resistance) OR TI=(Beta-ketothiolase Deficiency) OR TI=(Biotinidase Deficiency) OR TI=(Cardic Ion Channelopathies) OR TI=(Carnitine Deficiency) OR TI=(Castleman Disease) OR TI=(Charcot-Marie-Tooth Disease) OR TI=(Citrullinemia) OR TI=(Congenital Adrenal Hypoplasia) OR TI=(Congenital Hyperinsulinemic Hypoglycemia) OR TI=(Congenital Myasthenic Syndrome) OR TI=(Congenital Myotonia Syndrome) OR TI=(Non-Dystrophic Myotonia) OR TI=(Congenital Scoliosis) OR TI=(coronary Artery Ectasia) OR TI=(Diamond-Blackfan Anemia) OR TI=(Erdheim-Chester Disease) OR TI=(Fabry Disease) OR TI=(Familial Mediterranean Fever) OR TI=(Fanconi Anemia) OR TI=(Galactosemia) OR TI=(Gaucher Disease) OR TI=(Generalized Myasthenia Gravis) OR TI=(Gitelman Syndrome) OR TI=(Glutaric Acidemia Type I) OR TI=(Glutaric Acidemia Type I) OR TI=(Hemophilia) OR TI=(Hepatolenticular Degeneration) OR TI=(Wilson Disease) OR TI=(Hereditary Angioedema) OR TI=(Hereditary) OR TI=(Epidermolysis Bullosa) OR TI=(Hereditary Fructose Intolerance) OR TI=(Hereditary Hypomagnesemia) OR TI=(Hereditary Multi-infarct Dementia) OR TI=(Hereditary Spastic Paraplegia) OR TI=(Holocarboxylase Synthetase Deficiency) OR TI=(Homocysteinemia) OR TI=(Homozygous Hypercholesterolemia) OR TI=(Huntington Disease) OR TI=(Hyperornithinaemia-Hyperammonaemia-Homocitrullinuria Syndrome) OR TI=(Hyperphenylalaninemia) OR TI=(Hypophosphatasia) OR TI=(Hypophosphatemic Rickets) OR TI=(Idiopathic Cardiomyopathy) OR TI=(Idiopathic Hypogonadotropic Hypogonadism) OR TI=(Idiopathic Pulmonary Arterial Hypertension) OR TI=(Idiopathic Pulmonary Fibrosis) OR TI=(IgG4 related Disease) OR TI=(Inbornerrors of Bile Acid Synthesis) OR TI=(Isovaleric Acidemia) OR TI=(Kallmann Syndrome) OR TI=(Langerhans Cell Histiocytosis) results: 168150

2: TI=(Leber Hereditary Optic Neuropathy) OR TI=(Long Chain 3-hydroxyacyl-CoA) OR TI=(Dehydrogenase Deficiency) OR TI=(Lymphangioleiomyomatosis) OR TI=(Lysinuric Protein Intolerance) OR TI=(Lysosomal Acid Lipase Deficiency) OR TI=(Maple Syrup Urine Disease) OR TI=(Marfan Syndrome) OR TI=(McCune-Albright Syndrome) OR TI=(Medium Chain Acyl-CoA Dehydrogenase Deficiency) OR TI=(Methylmalonic Academia) OR TI=(Mitochodrial Encephalomyopathy) OR TI=(Mucopolysaccharidosi) OR TI=(Multifocal Motor Neuropathy) OR TI=(Multiple Acyl-CoA Dehydrogenase Deficiency) OR TI=(Multiple Sclerosis) OR TI=(Multiple System Atrophy) OR TI=(Myotonic Dystrophy) OR TI=(N-acetylglutamate Synthase Deficiency) OR TI=(Neonatal Diabetes Mellitus) OR TI=(Neuromyelitis Optica) OR TI=(Niemann-Pick Disease) OR TI=(Non-Syndromic Deafness) OR TI=(Noonan Syndrome) OR TI=(Ornithine Transcarbamylase Deficiency) OR TI=(Osteogenesis Imperfecta) OR TI=(Brittle Bone Disease) OR TI=(Young-onset Parkinson Disease) OR TI=(Early-onset Parkinson Disease) OR TI=(Paroxysmal Nocturnal Hemoglobinuria) OR TI=(Peutz-Jeghers Syndrome) OR TI=(Phenylketonuria) OR TI=(POEMS Syndrome) OR TI=(Porphyria) OR TI=(Prader-Willi Syndrome) OR TI=(Primary Combined Immune Deficiency) OR TI=(Primary Hereditary Dystonia) OR TI=(Primary Light Chain Amyloidosis) OR TI=(Progressive Familial Intrahepatic Cholestasis) OR TI=(Progressive Muscular Dystrophy) OR TI=(Propionic Acidemia) OR TI=(Pulmonary Alveolar Proteinosis) OR TI=(Pulmonary Cystic Fibrosis) OR TI=(Retinitis Pigmentosa) OR TI=(Retinoblastoma) OR TI=(Severe Congenital Neutropenia) OR TI=(Severe Myoclonic Epilepsy in Infancy) OR TI=(Dravet Syndrome) OR TI=(Sickle Cell Disease) OR TI=(Silver-Russell Syndrome) OR TI=(Sitosterolemia) OR TI=(Kennedy Disease) OR TI=(Spinal Muscular Atrophy) OR TI=(Spinocerebellar Ataxia) OR TI=(Systemic Sclerosis) OR TI=(Tetrahydrobiopterin Deficiency) OR TI=(Tuberous Sclerosis Complex) OR TI=(Tyrosinemia) OR TI=(Very Long Chain Acyl-CoA Dehydrogenase Deficiency) OR TI=(Williams Syndrome) OR TI=(Wiskott-Aldrich Syndrome) OR TI=(X-linked Agammaglobulinemia) OR TI=(X-linked Adrenoleukodystrophy) OR TI=(X-linked Lymphoproliferative Disease) results: 221650

3: TS=(Phytotherapy) OR TS=(Alternative medicine) OR TS=(Traditional medicine) OR TS=(medicinal plant) OR TS=(herb*) OR TS=(plant extract) OR TS=(plant preparation) OR TS=(natural compound*) OR TS=(Traditional Chinese medicine) OR TS=(Chinese drug) OR TS=(Chinese formul*) OR TS=(Chinese prescri*) OR TS=(Kampo medicine) OR TS=(Chinese materia medica) OR TS=(Japanese medicine) OR TS=(Japanese drug) OR TS=(Japanese formul*) OR TS=(Japanese prescri*) OR TS=(Korean medicine) OR TS=(Korean drug) OR TS=(Korean formul*) OR TS=(Korean prescri*) results: 774029

4: ((((TI=(randomized controlled trials)) OR TI=(clinical trials)) OR TI=(controlled trials)) OR TI=(double-blind)) OR TI=(clinical search) results: 322589

5: #1 AND #3 AND #4 results: 22

6: #2 AND #3 AND #4 results: 33

**4、Chinese National Knowledge Infrastructure Databased (CNKI):**

Subject = systemic sclerosis AND abstract = clinical + clinical efficacy + trial + randomized control + clinical research + efficacy AND subject = Chinese Medicine 101

Subject = idiopathic pulmonary fibrosis AND articles abstract = clinical + clinical efficacy + trial + randomized control + clinical research + efficacy AND main subject = idiopathic pulmonary fibrosis + clinical research + clinical observation + quality of life + quality of life + deficiency of both qi AND Yin + deficiency of both qi and Yin and subject = Chinese medicine

Results: 113

Subject = multiple sclerosis AND articles abstract = clinical + clinical efficacy + trial + randomized control + clinical research + efficacy AND Main subject = multiple sclerosis + clinical research + clinical observation AND subject = Traditional Chinese medicine + Integrated traditional Chinese and Western medicine

Results: 247

Subject = myasthenia gravis AND abstract = clinical + clinical efficacy + trial + clinical observation + clinical research + curative effect NOT title = eye muscle + experience + data mining + mechanism + progress + status + based on + acupuncture + cases + ideas + characteristics AND main subject = myasthenia gravis AND subject = traditional Chinese medicine + integrated traditional Chinese and Western medicine

Results: 335

Topic = Amyotrophic lateral sclerosis = neuronal disease AND abstract = clinical + clinical efficacy + trial + clinical observation + clinical research + efficacy NOT title = experience + data mining + mechanism + progress + status + based on + needle + case + idea + characteristics AND main topic = amyotrophic lateral sclerosis + amyotrophic lateral sclerosis + clinical efficacy observation AND Discipline = Traditional Chinese medicine + Integrated Chinese and Western Medicine

Results: 334

Theme = 21-hydroxylase deficiency + albinism + Alport syndrome + Angelman syndrome + arginase deficiency + Genner syndrome + atypical hemolytic uremic syndrome + autoimmune encephalitis + autoimmune hypophysitis + autoimmune insulin receptor disease + β-ketothiolase deficiency + biotinidase deficiency + cardiac ion channel disease + primary carnitine deficiency + Castleman disease + Peroneal muscular atrophy + citrullinemia + congenital adrenal hypoplasia + congenital hyperinsulinemic hypoglycemia + congenital myasthenic syndrome + Congenital myotonia + congenital scoliosis + coronary artery ectasia + congenital pure red cell aplasia + Erdheim-Chester disease + Fabry disease + familial Mediterranean fever + Fanconi anemia + galactosaemia + Gescher disease + Gitelman syndrome + congenital scoliosis + coronary artery ectasia + congenital pure red cell aplasia + Erdheim-Chester disease + Fabry disease + familial Mediterranean fever + Fanconi anemia + Gitelman syndrome + familial thalassemia Glutaric acidemia type I + glycogen storage disease + hepatolenticular degeneration + Wilson's disease + hereditary angiedema + hereditary epidermolysis bullosus + hereditary fructose intolerance + hereditary hypomagnesemia + hereditary multiple cerebral infarction dementia + hereditary spastic paraplegia + holocarbamase synthetase deficiency + Homocysteinemia + homozygous familial hypercholesterolemia + Huntington's disease + HHH syndrome + hyperphenylalaninemia + hypophosphatemia + hypophosphatemic rickets + idiopathic cardiomyopathy + idiopathic hypogonadotropic hypogonadism + idiopathic pulmonary hypertension + homocysteinemia + homozygous familial hypercholesterolemia + Huntington's disease + HHH syndrome + hyperphenylalaninemia + hypophosphatemia + hypophosphatemic rickets + idiopathic cardiomyopathy + idiopathic hypogonadotropic hypogonadism + idiopathic pulmonary hypertension + homocysteinemia Igg4-related disease + congenital bile acid synthesis disorder + isovaleric acidemia + Kallmann syndrome + Langerhans' histiocytosis + Leron syndrome + Leber hereditary optic neuropathy + long chain 3-hydroxyacyl-coa dehydrogenase deficiency + lymphangiomyomatosis + lysinuria protein intolerance + lysosomal acid lipase deficiency + maple syrup urine disease + Marfan syndrome + McCune-Albrigh syndrome + medium-chain acyl-coa dehydrogenase deficiency + methylmalonic acidemia + mitochondrial encephalomyopathy + mucopolysaccharidosis + multifocal motor neuropathy + multiple acyl-coa dehydrogenase deficiency Multiple system atrophy, myotonic dystrophy, N-acetylglutamate synthase deficiency, neonatal diabetes mellitus, neuromyelitis opticana, Niemann-Pick disease, non-syndromic deafness, Noonan syndrome, ornithine carbamoyltransferase deficiency, osteogenesis imperfecta, brittle bone disease Young-onset Parkinson's disease, early-onset Parkinson's disease, paroxysmal nocturnal hemoglobinuria, Peutzpah syndrome, phenylketonuria, POEMS syndrome, porphyria, Prader-Willi syndrome, primary combined immunodeficiency, and primary hereditary dystonia Primary light chain amyloidosis + progressive familial intrahepatic cholestasis + progressive muscular dystrophy + propionic acidemia + pulmonary alveolar proteinosis + pulmonary cystic fibrosis + retinitis pigmentosa + retinoblastoma + severe congenital agranulocytosis + severe myoclonic epilepsy of infancy + Dravet syndrome + Silver-Russell syndrome + sitosterolemia + spinal bulbar muscular atrophy + Kennedy's disease + spinal muscular atrophy + spinocerebellar ataxia + tetrahydrobiopterin deficiency + tuberous sclerosis + primary tyrosinemia + Very long chain acyl-coa dehydrogenase deficiency + Williams syndrome + eczema thrombocytopenia with immunodeficiency syndrome + X-linked agammagloglobulinemia + X-linked adrenoleukodystrophy + X-linked lymphohyperplasia = clinical + clinical efficacy + trial + clinical observation + clinical research + efficacy NOT 篇名= Experience + data mining + mechanism + progress + status + based + needles + examples + ideas + characteristics

Results: 0

**5、 SinoMed:**

(" Gaucher disease "[title: intelligence] OR "Gitelman syndrome "[title: intelligence] OR "glutaric acidemia type I "[title: intelligence] OR" glycogen storage disease "[title: intelligence] OR "hepatolenticular degeneration "[title: intelligence] OR" Wilson's disease "[title: intelligence] OR "Hereditary epidermolysis bullosa "[title: intelligence] OR" hereditary fructose intolerance "[title: intelligence] OR "hereditary hypomagnesemia "[title: intelligence] OR" hereditary multiple cerebral infarction dementia "[title: intelligence] OR "hereditary spastic paraplegia "[title: intelligence] OR "Pancarboxylase synthase deficiency "[title: intelligence] OR" homocysteinemia "[title: intelligence] OR "Huntington's disease "[title: intelligence] OR "HHH syndrome "[title: intelligence] OR" hyperphenylalaninemia "[title: intelligence] OR "hypophosphatasia "[title: intelligence] OR "hypophosphatemic rickets "[title: intelligence] OR" idiopathic cardiomyopathy "[title: intelligence] OR "idiopathic hypogonadotropic hypogonadism "[title: intelligence] OR" idiopathic pulmonary hypertension "[title: intelligence] OR "congenital bile acid synthesis disorder "[title: intelligence] OR "Isovaleric acidemia "[title: intelligence] OR" Kallmann syndrome "[title: intelligence] OR "Langerhans' histiocytosis "[title: intelligence] OR" Leron's syndrome "[title: intelligence] OR "Leber hereditary optic neuropathy "[title: intelligence] OR "Long chain 3-hydroxyacyl-coa dehydrogenase deficiency "[Title: intelligent]) AND(" Traditional Chinese medicine "[abstract: intelligent] OR" Chinese medicine formula granules "[abstract: intelligent] OR "decoction pieces "[abstract: intelligent] OR" traditional Chinese medicine treatment "[abstract: intelligent] OR "Chinese medicine prescription "[abstract: intelligent] OR "Particles" [abstract: smart] OR "prescription" [abstract: smart] OR "soup" [abstract: smart] OR [abstract: smart]) "drink", "clinical" [title: intelligent] OR "clinical efficacy of" [title: intelligent] OR "curative effect observation" [title: intelligent] the OR "Clinical curative effect observation [title: intelligent]" OR "clinical research" [title: intelligent])) NOT (" experience "[title: intelligent] OR" mechanism "[title: intelligent] OR [title: intelligent] OR progress" status quo "[title: intelligent] OR" thinking "[title: intelligent] the OR "Characteristics" [title: intelligent] OR "mining" [title: intelligent] OR "thinking" [title: intelligent] OR "feel" [title: intelligent] OR "explore" [title: intelligent]).

Results: 91

(" Lysinuria protein intolerance "[title: Intelligence] OR "Lysosomal acid lipase deficiency "[title: intelligence] OR" maple syrup urine disease "[title: intelligence] OR "Marfan syndrome "[title: intelligence] OR "McCune-Albrigh syndrome "[title: intelligence] OR "Medium-chain acyl-coa dehydrogenase deficiency" OR "methylmalonic acidemia" OR "mitochondrial encephalomyopathy" OR "multifocal motor neuropathy" OR "multiple acyl-coa dehydrogenase deficiency" OR "Multiple system atrophy "[title: intelligence] OR" myotonic dystrophy "[title: intelligence] OR "N-acetylglutamate synthase deficiency "[title: intelligence] OR" neonatal diabetes "[title: intelligence] OR "neuromyelitis optica "[title: intelligence] OR" Niemann-Pick disease "[title: intelligence] OR "Noonan syndrome "[title: Intelligence] OR "ornithine transcarbamylase deficiency "[title: intelligence] OR" osteogenesis imperfecta "[title: intelligence] OR "brittle bone disease "[title: intelligence] OR" young onset Parkinson's disease "[title: intelligence] OR "early onset Parkinson's disease "[title: intelligence] OR "paroxysmal nocturnal hemoglobinuria "[title: intelligence] OR" Peutzpah syndrome "[title: intelligence] OR "phenylketonuria "[title: intelligence] OR" porphyria "[title: intelligence] OR "Prader-Willi syndrome "[title: intelligence] OR "Primary combined immunodeficiency "[title: intelligence] OR" primary hereditary dystonia "[title: intelligence] OR "primary light chain amyloidosis "[title: intelligence] OR" progressive familial intrahepatic cholestasis "[title: intelligence] OR "progressive muscular dystrophy "[title: intelligence] OR "Propionic acidemia "[title: intelligence] OR" pulmonary cystic fibrosis "[title: intelligence] OR "retinitis pigmenti "[title: intelligence] OR" retinoblastoma "[title: intelligence] OR "severe congenital agranulocytosis "[title: intelligence]) AND(" traditional Chinese medicine "[abstract: Intelligence] OR "Traditional Chinese medicine formula particles" [abstract: smart] OR "slices" [abstract: smart] OR "treatment of traditional Chinese medicine" [abstract: smart] OR "prescription" [abstract: smart] OR "particles" [abstract: smart] OR "prescription" [abstract: smart] OR "soup" OR [abstract: smart] [abstract: smart]) "drink", "clinical" [abstract: smart] OR "clinical efficacy of" [abstract: smart] OR "curative effect observation" [abstract: smart] OR "clinical curative effect observation" [abstract: smart] OR "clinical research" [abstract: smart])) NOT (" experience "[title: intelligent] the OR "Mechanism" [title: intelligent] OR "progress" [title: intelligent] OR [title: intelligent] "OR" thinking "[title: intelligent] OR" features "[title: intelligent] OR" mining "[title: intelligent] OR" thinking "[title: intelligent] OR" feel "[title: intelligent] the OR "Discussion "[title: Intelligence])

Results: 166

(" Infantile severe myoclonic epilepsy "[title: intelligence] OR "Dravet syndrome "[title: intelligence] OR "Silver-Russell syndrome "[title: intelligence] OR "sitosterolemia "[title: intelligence] OR" spinal muscular atrophy "[title: intelligence] OR "Spinocerebellar ataxia "[title: intelligence] OR" tetrahydrobiopterin deficiency "[title: intelligence] OR "tuberous sclerosis "[title: intelligence] OR" primary tyrosinemia "[title: intelligence] OR "very long chain acyl-coa dehydrogenase deficiency "[title: intelligence] OR "Wilms syndrome "[title: intelligence] OR" eczema thrombocytopenia with immunodeficiency syndrome "[title: intelligence] OR "X-linked agammaglobulinemia "[title: intelligence] OR" X-linked adrenoleukodystrophy "[title: intelligence] OR "X-linked lymphoproliferative disease "[title: intelligence] OR "Erdheim-Chester disease "[title: intelligence] OR "citrullinemia "[title: intelligence] OR" hereditary angioedema "[title: intelligence] OR "homozygous familial hypercholesterolemia "[title: intelligence] OR" IgG4-related disease "[title: intelligence] OR "Lymphatic leiomyomatosis "[title: Intelligence] OR" mucopolysaccharidosis "[title: intelligence] OR "non-syndromic deafness "[title: intelligence] OR "POEMS syndrome "[title: intelligence] OR" pulmonary alveolar proteinosis "[title: intelligence] OR "spinal bulbar muscular atrophy "[title: intelligence] OR "Kennedy disease" [title: intelligent]) AND (" Chinese medicine "[abstract: smart] OR" traditional Chinese medicine formula particles [abstract: smart] "OR" slices "[abstract: smart] OR" treatment of traditional Chinese medicine "[abstract: smart] OR" prescription "[abstract: smart] OR" particles "[abstract: smart] the OR "Prescription" [abstract: smart] OR "soup" [abstract: smart] OR [abstract: smart]) "drink", "clinical" [abstract: smart] OR "clinical efficacy of" [abstract: smart] OR "curative effect observation" [abstract: smart] OR "clinical curative effect observation" [abstract: smart] the OR "Clinical research" [abstract: smart])) NOT (" experience "[title: intelligent] OR" mechanism "[title: intelligent] OR [title: intelligent] OR progress" status quo "[title: intelligent] OR" thinking "[title: intelligent] OR" features "[title: intelligent] the OR "Dig" [title: intelligent] OR "thinking" [title: intelligent] OR "feel" [title: intelligent] OR "explore" [title: intelligent]).

Results: 65

((" systemic sclerosis "[title: intelligent] OR "idiopathic pulmonary fibrosis "[title: intelligent] OR" multiple sclerosis "[title: intelligent] OR "myasthenia gravis "[title: intelligent] OR" amyotrophic lateral sclerosis "[title: intelligent]) AND(" Chinese medicine "[abstract: Intelligent] OR "Traditional Chinese medicine formula particles" [abstract: smart] OR "slices" [abstract: smart] OR "treatment of traditional Chinese medicine" [abstract: smart] OR "prescription" [abstract: smart] OR "particles" [abstract: smart] OR "prescription" [abstract: smart] OR "soup" OR [abstract: smart] [abstract: smart]) "drink", "clinical" [title: intelligent] OR "clinical efficacy of" [title: intelligent] OR "curative effect observation" [title: intelligent] OR "clinical curative effect observation [title: intelligent]" OR "clinical research" [title: intelligent])) NOT (" experience "[title: intelligent] the OR "Mechanism" [title: intelligent] OR "progress" [title: intelligent] OR [title: intelligent] "OR" thinking "[title: intelligent] OR" features "[title: intelligent] OR" mining "[title: intelligent] OR" thinking "[title: intelligent] OR" feel "[title: intelligent] the OR "Discussion "[title: Intelligence])

Results:200

**6、 Wanfang:**

Title OR key words :(systemic sclerosis) and Subject :(Chinese medicine OR Chinese medicine OR Chinese medicine formula granules OR herbal pieces OR Chinese medicine treatment OR Chinese medicine OR granules OR prescription OR decoction OR drink) and title :(clinical OR clinical efficacy OR test or efficacy observation OR clinical research OR efficacy OR clinical observation) not title :(experience OR mechanism OR progress OR status quo OR ideas OR characteristics OR mining OR thinking)

Results: 28

Title OR key words :(idiopathic pulmonary fibrosis) and subject :(Chinese medicine OR Chinese medicine OR Chinese medicine formula granules OR herbal pieces OR Chinese medicine treatment OR Chinese medicine OR granules OR prescription OR decoction OR drink) and title :(clinical OR clinical efficacy OR test or efficacy observation OR clinical research OR efficacy OR clinical observation) not title :(experience OR mechanism OR progress OR status quo OR ideas OR characteristics OR mining OR thinking)

Results: 126

Title OR key words :(multiple sclerosis) and Subject :(Chinese medicine OR Chinese medicine OR Chinese medicine formula granules OR herbal pieces OR Chinese medicine treatment OR Chinese medicine OR granules OR prescription OR decoction OR drink) and title :(clinical OR clinical efficacy OR test or efficacy observation OR clinical research OR efficacy OR clinical observation) not title :(experience OR mechanism OR progress OR status quo OR ideas OR characteristics OR mining OR thinking)

Results: 66

Title OR key words :(Myasthenia gravis) and subject :(Chinese medicine OR Chinese medicine OR Chinese medicine formula granules OR herbal pieces OR Chinese medicine treatment OR Chinese medicine OR granules OR prescription OR decoction OR drink) and title :(clinical OR clinical efficacy OR test or efficacy observation OR clinical research OR efficacy OR clinical observation) not title :(experience OR mechanism OR progress OR status quo OR ideas OR characteristics OR mining OR thinking)

Results: 224

Title OR key words :(Amyotrophic lateral sclerosis) and Subject :(Chinese medicine OR Chinese medicine OR Chinese medicine formula granules OR herbal pieces OR Chinese medicine treatment OR Chinese medicine OR granules OR formula OR decoction OR decoction) and title :(clinical OR clinical efficacy OR test or efficacy observation OR clinical research OR efficacy OR clinical observation) not title :(experience OR mechanism OR progress OR status quo OR ideas OR characteristics OR mining OR thinking)

Results: 55

Title OR key words :(21-hydroxylase deficiency OR albinism OR Alport syndrome OR Angelman syndrome OR arginase deficiency OR Genna syndrome OR atypical hemolytic uremia or autoimmune encephalitis) and subject :(Traditional Chinese Medicine) OR Chinese medicine OR Chinese medicine formula granules OR herbal pieces OR Chinese medicine treatment OR Chinese medicine prescription OR granules OR prescription OR decoction OR decoction) and title :(clinical OR clinical efficacy OR test or efficacy observation OR clinical research OR efficacy OR clinical observation) not title :(experience OR mechanism OR progress OR status quo OR ideas OR characteristics OR mining OR thinking)

Results: 2

Title OR key words :(autoimmune hypophysitis OR autoimmune insulin receptor disease OR β-ketothiolase deficiency OR biotinidase deficiency OR cardiac ion channel disease OR primary carnitine deficiency OR Castleman disease OR Charcot-Marie-tooth disease) and subject :(Traditional Chinese medicine or Traditional Chinese medicine) OR Chinese medicine formula granules OR herbal pieces OR Chinese medicine treatment OR Chinese medicine formula OR granules OR prescription OR decoction OR decoction) and title :(clinical OR clinical efficacy OR test or efficacy observation OR clinical research OR efficacy OR clinical observation) not title :(experience OR mechanism OR progress OR status quo OR ideas OR characteristics OR mining OR thinking)

Results: 4

Title OR key words :(Fabry disease OR familial Mediterranean fever OR Fanconi anemia OR galactosaemia OR Gaucher disease OR Gitelman syndrome OR glutaric acidemia type I OR glycogen storage disease OR hepatolenticular degeneration OR Wilson's disease) and subject :(TCM OR Chinese medicine OR Chinese medicine formula granules OR herbal pieces OR Chinese medicine treatment OR Chinese medicine formula OR granules OR prescription OR decoction OR decoction) and title :(clinical OR clinical efficacy OR test or efficacy observation OR clinical research OR efficacy OR clinical observation) not title :(experience OR mechanism OR progress OR status quo OR ideas OR characteristics OR mining OR thinking)

Results: 90

Title OR key words :(hereditary epidermolysis bullosamina OR hereditary fructose intolerance OR hereditary hypomagnesemia OR hereditary multiple cerebral infarction dementia OR hereditary spastic paraplegia OR holocarboxylase synthase deficiency OR homocysteinemia) and subject :(Traditional Chinese medicine OR Chinese medicine OR OR) Chinese medicine formula granule OR decoction OR Chinese medicine treatment OR Chinese medicine prescription OR granule OR prescription OR decoction OR decoction) and title :(clinical OR clinical efficacy OR test or efficacy observation OR clinical research OR efficacy OR clinical observation) not title :(experience OR mechanism OR progress OR status quo OR ideas OR characteristics OR mining OR thinking)

Results: 57

Title OR key words :(Huntington's disease OR HHH syndrome OR hyperphenylalaninemia OR hypophosphatemia OR hypophosphatemic rickets OR idiopathic cardiomyopathy OR idiopathic hypogonadotropic hypogonadism OR idiopathic pulmonary hypertension) and subject :(Traditional Chinese medicine or Chinese medicine) OR Chinese medicine formula granules OR herbal pieces OR Chinese medicine treatment OR Chinese medicine prescription OR granules OR prescription OR decoction OR decoction) and title :(clinical OR clinical efficacy OR trial OR efficacy observation OR clinical research OR efficacy OR clinical observation) not title :(Experience OR mechanism OR progress OR status OR ideas OR characteristics OR mining OR thinking)

Results: 6

Title OR key words :(congenital bile acid synthesis disorder OR isovaleric acidemia OR Kalman syndrome OR Langerhans histiocytosis OR Leren syndrome OR Leber hereditary optic neuropathy OR long chain 3-hydroxyacyl-coa dehydrogenase deficiency) and subject :(Traditional Chinese medicine OR Chinese medicine OR) Chinese medicine formula granules OR herbal pieces OR Chinese medicine treatment OR Chinese medicine OR granules OR prescription OR decoction OR decoction) and title :(clinical OR clinical efficacy OR trial OR efficacy observation OR clinical research OR efficacy OR clinical observation) not title :(Experience OR mechanism OR progress OR status OR ideas OR characteristics OR mining OR thinking)

Results: 8

Title OR key words :(lysinuria protein intolerance OR lysosomal acid lipase deficiency OR maple urine disease OR Marfan syndrome OR McCune-Albrigh syndrome OR medium-chain acyl-coa dehydrogenase deficiency OR methylmalonic acidemia or mitochondrial brain muscle) and subject :(Traditional Chinese Medicine) OR Chinese medicine OR Chinese medicine formula granules OR herbal pieces OR Chinese medicine treatment OR Chinese medicine prescription OR granules OR prescription OR decoction OR decoction) and title :(clinical OR clinical efficacy OR test or efficacy observation OR clinical research OR efficacy OR clinical observation) not title :(experience OR mechanism OR progress OR status quo OR ideas OR characteristics OR mining OR thinking)

Results: 14

Title OR key words :(Multifocal motor neuropathy OR multiple acyl-coenzyme A dehydrogenase deficiency OR multiple system atrophy OR myotonic dystrophy OR N-acetylglutamate synthase deficiency OR neonatal diabetes OR neuromyelitis optica OR Niemann-Pick disease) and subject :(Traditional Chinese medicine or Chinese medicine) OR Chinese medicine formula granules OR herbal pieces OR Chinese medicine treatment OR Chinese medicine prescription OR granules OR prescription OR decoction OR decoction) and title :(clinical OR clinical efficacy OR trial OR efficacy observation OR clinical research OR efficacy OR clinical observation) not title :(Experience OR mechanism OR progress OR status OR ideas OR characteristics OR mining OR thinking)

Results: 41

Title OR key words :(Noonan syndrome OR ornithine carbamoyltransferase deficiency OR osteogenesis imperfecta OR brittle bone disease OR young-onset Parkinson's disease OR early-onset Parkinson's disease OR paroxysmal nocturnal hemoglobinuria OR Peutz syndrome or phenylketonuria) and subject :(TCM) OR Chinese medicine OR Chinese medicine formula granules OR herbal pieces OR Chinese medicine treatment OR Chinese medicine prescription OR granules OR prescription OR decoction OR decoction) and title :(clinical OR clinical efficacy OR test or efficacy observation OR clinical research OR efficacy OR clinical observation) not title :(experience OR mechanism OR progress OR status quo OR ideas OR characteristics OR mining OR thinking)

Results: 50

Title OR key words :(Porphyria OR Prader-Willi syndrome OR primary combined immunodeficiency OR primary hereditary dystonia OR primary light chain amyloidosis OR progressive familial intrahepatic cholestasis OR progressive muscular dystrophy or propionic acidemia) and subject :(TCM) OR Chinese medicine OR Chinese medicine formula granules OR herbal pieces OR Chinese medicine treatment OR Chinese medicine prescription OR granules OR prescription OR decoction OR decoction) and title :(clinical OR clinical efficacy OR test or efficacy observation OR clinical research OR efficacy OR clinical observation) not title :(experience OR mechanism OR progress OR status quo OR ideas OR characteristics OR mining OR thinking)

Results: 37

Title OR key words :(pulmonary cystic fibrosis OR retinitis pigmentosus OR retinoblastoma OR severe congenital agranulocytosis OR infantile severe myoclonic epilepsy OR Dravet syndrome OR Silver-Russell syndrome or sitosterolemia) and subject :(TCM) OR Chinese medicine OR Chinese medicine formula granules OR herbal pieces OR Chinese medicine treatment OR Chinese medicine prescription OR granules OR prescription OR decoction OR decoction) and title :(clinical OR clinical efficacy OR test or efficacy observation OR clinical research OR efficacy OR clinical observation) not title :(experience OR mechanism OR progress OR status quo OR ideas OR characteristics OR mining OR thinking)

Results: 58

Title OR key words :(spinal muscular atrophy OR spinocerebellar ataxia OR tetrahydrobiopterin deficiency OR tuberous sclerosis OR primary tyrosinemia OR very long chain acyl-coa dehydrogenase deficiency) and subject :(Traditional Chinese medicine OR Chinese medicine OR Chinese medicine formula granules OR decoction pieces OR) Chinese medicine treatment OR Chinese medicine prescription OR granule OR prescription OR decoction OR decoction) and title :(clinical OR clinical efficacy OR trial OR efficacy observation OR clinical research OR efficacy OR clinical observation) not title :(experience OR mechanism OR progress OR status quo OR thinking OR characteristic OR digging OR thinking)

Results: 6

Title OR key words :(Erdheim-Chester disease OR citrullinemia OR hereditary angioedema OR homozygous familial hypercholesterolemia OR IgG4-related disease OR lymphangioleiomyomatosis) and subject :(Traditional Chinese Medicine OR Chinese medicine OR Chinese medicine formula granules or herbal pieces) OR TCM treatment OR Chinese medicine prescription OR granule OR prescription OR decoction OR decoction) and title :(Clinical OR clinical efficacy OR trial OR efficacy observation OR clinical research OR efficacy OR clinical observation) not title :(experience OR mechanism OR progress OR Current situation OR ideas OR characteristics OR digging OR thinking)

Results: 3

Title OR key words :(mucopolysaccharidosis OR non-syndromic deafness OR POEMS syndrome OR pulmonary alveolar proteinosis OR spinal bulbar muscular atrophy OR Kennedy's disease) and subject :(Traditional Chinese medicine OR Chinese medicine OR Chinese medicine OR Chinese medicine formula granules OR decoction pieces OR Chinese medicine treatment OR Chinese medicine prescription OR) Granule OR prescription OR decoction OR decoction) and title :(Clinical OR clinical efficacy OR trial OR efficacy observation OR clinical research OR efficacy OR clinical observation) not title :(experience OR mechanism OR progress OR status quo OR ideas OR characteristics OR Dig OR think)

Results: 14

Title OR key words :(congenital adrenal hypoplasia OR congenital hyperinsulinemic hypoglycemia OR congenital myasthenic syndrome OR congenital myotonia OR congenital scoliosis OR coronary artery ectasia OR congenital pure red cell aplasia) and subject :(Traditional Chinese medicine OR Chinese medicine OR) Chinese medicine formula granule OR decoction OR Chinese medicine treatment OR Chinese medicine prescription OR granule OR prescription OR decoction OR decoction) and title :(clinical OR clinical efficacy OR trial OR efficacy observation OR clinical research OR efficacy OR clinical observation) not title :(Experience OR Mechanism OR progress OR status quo OR ideas OR characteristics OR mining OR thinking)

Results: 0

Title OR key words :(Williams syndrome OR eczema thrombocytopenia with immunodeficiency syndrome OR X-linked agammaglobulinemia OR X-linked adrenoleukodystrophy OR X-linked lymphohyperplasia) and subject :(Traditional Chinese medicine OR Chinese medicine OR Chinese medicine formula granules OR decoction pieces OR) Chinese medicine treatment OR Chinese medicine prescription OR granule OR prescription OR decoction OR decoction) and title :(clinical OR clinical efficacy OR trial OR efficacy observation OR clinical research OR efficacy OR clinical observation) not title :(experience OR mechanism OR progress OR status quo OR ideas OR characteristics OR mining OR thinking

Results: 0

**7、 VIP:**

T=(21-hydroxylase deficiency OR albinism OR Alport syndrome OR Angelman syndrome OR Angelman syndrome OR arginase deficiency OR Genner syndrome OR atypical hemolytic uremic syndrome OR autoimmune encephalitis OR autoimmune hypophysitis OR Autoimmune insulin receptor disease OR β-ketothiolase deficiency OR biotinidase deficiency OR cardiac ion channel disease OR primary carnitine deficiency OR Castleman disease OR Charcot-Marie-tooth disease OR citrullinemia OR congenital adrenal hypoplasia OR "Congenital hyperinsulinemic hypoglycemia OR congenital myasthenic syndrome OR congenital myotonia OR congenital scoliosis OR coronary artery ectasia OR congenital pure red cell aplasia OR Erdheim-Chester disease OR Fabry disease OR familial Mediterranean fever OR Fanconi anemia OR galactosaemia OR Gaucher disease OR Gitelman syndrome OR glutaric acidemia type I OR glycogen storage disease OR hepatolenticular degeneration OR Wilson's disease OR hereditary angioedema OR hereditary epidermolysis bullosa OR hereditary fructose intolerance OR "Hereditary hypomagnesemia OR hereditary multiple cerebral infarction dementia OR hereditary spastic paraplegia OR pancarboxylase synthase deficiency OR homocysteinemia OR homozygous familial hypercholesterolemia OR Huntington's disease OR HHH syndrome OR hyperphenylalaninemia OR hypophosphatemia. OR hypophosphatemic rickets) AND M=(Chinese medicine OR Chinese medicine formula granules OR herbal pieces OR Chinese medicine treatment OR Chinese medicine OR granules OR prescription OR decoction OR drink) AND T=(clinical OR clinical efficacy OR efficacy observation OR trial OR clinical study OR efficacy OR clinical observation) NOT T=(experience OR mechanism OR progress OR status quo OR ideas OR characteristics OR mining OR thinking OR experience OR exploration OR evolution of OR) + Add search title in results = T=(21-hydroxylase deficiency OR albinism OR Alport syndrome OR Angelman syndrome OR Angelman syndrome OR arginase deficiency OR Genner syndrome OR atypical hemolytic uremic syndrome OR autoimmune encephalitis OR autoimmune hypophylitis OR autoimmune insulin receptor disease OR β-ketothiolase deficiency OR biotinidase deficiency OR cardiac ion channel disease OR primary carnitine deficiency OR Castleman disease OR Peroneal muscular atrophy OR citrullinemia OR congenital adrenal hypoplasia OR congenital hyperinsulinemic hypoglycemia OR congenital myasthenic syndrome OR congenital myotonia OR congenital scoliosis OR coronary artery ectasia OR congenital pure red cell aplasia OR Erdheim-Chester disease OR Fabry disease OR familial Mediterranean fever OR Fanconi anemia OR galactosaemia OR Gaulcher disease OR Gitelman syndrome OR glutaric acidemia type I OR glycogen storage disease OR hepatolenticular degeneration OR Wilson's disease OR hereditary angioedema OR hereditary epidermolysis bullosa OR hereditary fructose intolerance OR hereditary hypomagnesemia OR hereditary multiple cerebral infarction dementia OR hereditary spastic paraplegia OR Pancarboxylase synthase deficiency OR homocysteinemia OR homozygous familial hypercholesterolemia OR Huntington's disease OR the HHH syndrome OR hyperphenylalaninemia OR hypophosphatemia OR hypophosphatemic rickets)

Results: 0

T=(Idiopathic cardiomyopathy OR idiopathic hypogonadotropic hypogonadism OR idiopathic pulmonary hypertension OR IgG4-related disease OR congenital bile acid synthesis disorder OR isovaleric acidemia OR Kallman's syndrome OR Langerhans' histiocytosis OR Leron's syndrome OR Leber hereditary optic neuropathy OR long-chain 3-hydroxyacyl-coa dehydrogenase deficiency OR lymphangioleiomyomatosis OR lysinuria protein intolerance OR lysosomal acid lipase deficiency OR maple urine disease OR Marfan syndrome OR McCune-Albrigh syndrome OR OR Medium-chain acyl-coa dehydrogenase deficiency OR methylmalonic acidemia OR mitochondrial encephalomyopathy OR mucopolysaccharidosis OR multifocal motor neuropathy OR multiple acyl-coa dehydrogenase deficiency OR multiple system atrophy OR myotonic dystrophy OR N-acetylglutamate synthetase deficiency OR Neonatal diabetes mellitus OR neuromyelitis optica OR Niemann-Pick disease) AND M=(Chinese medicine OR Chinese medicine formula granules OR herbal pieces OR Chinese medicine treatment OR Chinese medicine OR granules OR prescription OR decoction OR drink) AND T=(clinical OR clinical efficacy OR efficacy observation OR OR) Trial OR clinical study OR efficacy OR clinical observation) NOT T=(experience OR mechanism OR progress OR status OR ideas OR characteristics OR mining OR thinking OR experience OR exploration OR evolution of OR)

Results: 74

T=(non-syndromic deafness OR Noonan syndrome OR ornithine carbamoyltransferase deficiency OR osteogenesis imperfecta OR brittle bone disease OR young-onset Parkinson's disease OR early-onset Parkinson's disease OR paroxysmal nocturnal hemoglobinuria OR Peutz syndrome OR phenylketonuria OR POEMS syndrome OR porphyria OR Prader-Willi syndrome OR primary combined immunodeficiency OR primary hereditary dystonia OR primary light chain amyloidosis OR progressive familial intrahepatic cholestasis OR progressive muscular dystrophy OR propionic acidemia OR Pulmonary alveolar proteinosis OR pulmonary cystic fibrosis OR retinitis pigmentosa OR retinoblastoma OR severe congenital agranulocytosis OR severe myoclonic epilepsy of infants OR Dravet syndrome OR Silver-Russell syndrome OR sitosterolemia OR OR myelomedulbar muscular atrophy OR Kennedy's disease OR spinal muscular atrophy OR spinocerebellar ataxia OR tetrahydrobiopterin deficiency OR tuberous sclerosis OR primary tyrosinemia OR very-long-chain acyl-coa dehydrogenase deficiency OR Williams syndrome Eczema thrombocytopenia with immunodeficiency syndrome OR X-linked agammaglobulinemia OR X-linked adrenoleukodystrophy OR X-linked lymphohyperplasia) AND M=(Chinese medicine OR Chinese medicine formula granules OR decoction pieces OR Chinese medicine treatment OR Chinese medicine formula OR granules OR prescription OR or prescription OR Decoction OR drink) AND T=(clinical OR clinical efficacy OR efficacy observation OR trial OR clinical research OR efficacy OR clinical observation) NOT T=(experience OR mechanism OR progress OR status quo OR ideas OR characteristics OR mining OR thinking OR experience OR to explore the evolution of OR)

Results: 62

T=(systemic sclerosis OR idiopathic pulmonary fibrosis OR multiple sclerosis OR myasthenia gravis OR amyotrophic lateral sclerosis) AND M=(Chinese medicine OR Chinese medicine formula granules OR herbal pieces OR Chinese medicine treatment OR Chinese medicine OR granules OR prescriptions OR decoction OR decoction) AND T=(clinical OR clinical efficacy OR efficacy observation OR trial OR clinical research OR efficacy OR clinical observation) NOT T=(experience OR mechanism OR progress OR status quo OR ideas OR characteristics OR mining OR thinking OR experience OR exploration OR evolution)

Results: 149

**8、CiNii**

(‘‘Leber Hereditary Optic Neuropathy’‘ OR ‘‘Long Chain 3-hydroxyacyl-CoA’‘ OR ‘‘Dehydrogenase Deficiency’‘ OR ‘‘Lymphangioleiomyomatosis’‘ OR ‘‘Lysinuric Protein Intolerance’‘ OR ‘‘Lysosomal Acid Lipase Deficiency’‘ OR ‘‘Maple Syrup Urine Disease’‘ OR ‘‘Marfan Syndrome’‘ OR ‘‘McCune-Albright Syndrome’‘ OR ‘‘Medium Chain Acyl-CoA Dehydrogenase Deficiency’‘ OR ‘‘Methylmalonic Academia’‘ OR ‘‘Mitochodrial Encephalomyopathy’‘ OR ‘‘Mucopolysaccharidosi’‘ OR ‘‘Multifocal Motor Neuropathy’‘ OR ‘‘Multiple Acyl-CoA Dehydrogenase Deficiency’‘ OR ‘‘Multiple Sclerosis’‘ OR ‘‘Multiple System Atrophy’‘ OR ‘‘Myotonic Dystrophy’‘ OR ‘‘N-acetylglutamate Synthase Deficiency’‘ OR ‘‘Neonatal Diabetes Mellitus’‘ OR ‘‘Neuromyelitis Optica’‘ OR ‘‘Niemann-Pick Disease’‘ OR ‘‘Non-Syndromic Deafness’‘ OR ‘‘Noonan Syndrome’‘ OR ‘‘Ornithine Transcarbamylase Deficiency’‘ OR ‘‘Osteogenesis Imperfecta’‘ OR ‘‘Brittle Bone Disease’‘ OR ‘‘Young-onset Parkinson Disease’‘ OR ‘‘Early-onset Parkinson Disease’‘ OR ‘‘Paroxysmal Nocturnal Hemoglobinuria’‘ OR ‘‘Peutz-Jeghers Syndrome’‘ OR ‘‘Phenylketonuria’‘ OR ‘‘POEMS Syndrome’‘ OR ‘‘Porphyria’‘ OR ‘‘Prader-Willi Syndrome’‘ OR ‘‘Primary Combined Immune Deficiency’‘ OR ‘‘Primary Hereditary Dystonia’‘ OR ‘‘Primary Light Chain Amyloidosis’‘ OR ‘‘Progressive Familial Intrahepatic Cholestasis’‘ OR ‘‘Progressive Muscular Dystrophy’‘ OR ‘‘Propionic Acidemia’‘ OR ‘‘Pulmonary Alveolar Proteinosis’‘ OR ‘‘Pulmonary Cystic Fibrosis’‘ OR ‘‘Retinitis Pigmentosa’‘ OR ‘‘Retinoblastoma’‘ OR ‘‘Severe Congenital Neutropenia’‘ OR ‘‘Severe Myoclonic Epilepsy in Infancy’‘ OR ‘‘Dravet Syndrome’‘ OR ‘‘Sickle Cell Disease’‘ OR ‘‘Silver-Russell Syndrome’‘ OR ‘‘Sitosterolemia’‘ OR ‘‘Kennedy Disease’‘ OR ‘‘Spinal Muscular Atrophy’‘ OR ‘‘Spinocerebellar Ataxia’‘ OR ‘‘Systemic Sclerosis’‘ OR ‘‘Tetrahydrobiopterin Deficiency’‘ OR ‘‘Tuberous Sclerosis Complex’‘ OR ‘‘Tyrosinemia’‘ OR ‘‘Very Long Chain Acyl-CoA Dehydrogenase Deficiency’‘ OR ‘‘Williams Syndrome’‘ OR ‘‘Wiskott-Aldrich Syndrome’‘ OR ‘‘X-linked Agammaglobulinemia’‘ OR ‘‘X-linked Adrenoleukodystrophy’‘ OR ‘‘X-linked Lymphoproliferative Disease’‘) AND ('’medicinal plant’‘ OR ‘‘japanese medicine’‘ OR ‘‘kampo medicine’‘ OR ‘‘traditional medicine’‘ OR ‘‘herbaceous agent’‘ OR ‘‘plant extract’‘ OR ‘‘alternative medicine’‘ OR ‘‘traditional medicine'’) AND (‘’clinical trials’’OR ‘’randomized clinical trial’’ OR ‘’placebo’’) 0

(‘‘21-Hydroxylase Deficiency’‘ OR ‘‘Albinism’‘ OR ‘‘Alport Syndrome’‘ OR ‘‘Amyotrophic Lateral Sclerosis’‘ OR ‘‘Angelman Syndrome’‘ OR ‘‘Arginase Deficiency’‘ OR ‘‘Asphyxiating Thoracic Dystrophy’‘ OR ‘‘Jeune Syndrome’‘ OR ‘‘Atypical Hemolytic Uremic Syndrome’‘ OR ‘‘Autoimmune Encephalitis’‘ OR ‘‘Autoimmune Hypophysitis’‘ OR ‘‘Autoimmune Insulin Receptopathy’‘ OR ‘‘Type B insulin resistance’‘ OR ‘‘Beta-ketothiolase Deficiency’‘ OR ‘‘Biotinidase Deficiency’‘ OR ‘‘Cardic Ion Channelopathies’‘ OR ‘‘Carnitine Deficiency’‘ OR ‘‘Castleman Disease’‘ OR ‘‘Charcot-Marie-Tooth Disease’‘ OR ‘‘Citrullinemia’‘ OR ‘‘Congenital Adrenal Hypoplasia’‘ OR ‘‘Congenital Hyperinsulinemic Hypoglycemia’‘ OR ‘‘Congenital Myasthenic Syndrome’‘ OR ‘‘Congenital Myotonia Syndrome’‘ OR ‘‘Non-Dystrophic Myotonia’‘ OR ‘‘Congenital Scoliosis’‘ OR ‘‘coronary Artery Ectasia’‘ OR ‘‘Diamond-Blackfan Anemia’‘ OR ‘‘Erdheim-Chester Disease’‘ OR ‘‘Fabry Disease’‘ OR ‘‘Familial Mediterranean Fever’‘ OR ‘‘Fanconi Anemia’‘ OR ‘‘Galactosemia’‘ OR ‘‘Gaucher Disease’‘ OR ‘‘Generalized Myasthenia Gravis’‘ OR ‘‘Gitelman Syndrome’‘ OR ‘‘Glutaric Acidemia Type I’‘ OR ‘‘Glutaric Acidemia Type I’‘ OR ‘‘Hemophilia’‘ OR ‘‘Hepatolenticular Degeneration’‘ OR ‘‘Wilson Disease’‘ OR ‘‘Hereditary Angioedema’‘ OR ‘‘Hereditary’‘ OR ‘‘Epidermolysis Bullosa’‘ OR ‘‘Hereditary Fructose Intolerance’‘ OR ‘‘Hereditary Hypomagnesemia’‘ OR ‘‘Hereditary Multi-infarct Dementia’‘ OR ‘‘Hereditary Spastic Paraplegia’‘ OR ‘‘Holocarboxylase Synthetase Deficiency’‘ OR ‘‘Homocysteinemia’‘ OR ‘‘Homozygous Hypercholesterolemia’‘ OR ‘‘Huntington Disease’‘ OR ‘‘Hyperornithinaemia-Hyperammonaemia-Homocitrullinuria Syndrome’‘ OR ‘‘Hyperphenylalaninemia’‘ OR ‘‘Hypophosphatasia’‘ OR ‘‘Hypophosphatemic Rickets’‘ OR ‘‘Idiopathic Cardiomyopathy’‘ OR ‘‘Idiopathic Hypogonadotropic Hypogonadism’‘ OR ‘‘Idiopathic Pulmonary Arterial Hypertension’‘ OR ‘‘Idiopathic Pulmonary Fibrosis’‘ OR ‘‘IgG4 related Disease’‘ OR ‘‘Inbornerrors of Bile Acid Synthesis’‘ OR ‘‘Isovaleric Acidemia’‘ OR ‘‘Kallmann Syndrome’‘ OR ‘‘Langerhans Cell Histiocytosis’‘) AND ('’medicinal plant’‘ OR ‘‘japanese medicine’‘ OR ‘‘kampo medicine’‘ OR ‘‘traditional medicine’‘ OR ‘‘herbaceous agent’‘ OR ‘‘plant extract’‘ OR ‘‘alternative medicine’‘ OR ‘‘traditional medicine'’) AND (‘’clinical trials’’OR ‘’randomized clinical trial’’ OR ‘’placebo’’).

Results: 0
