# Supplementary material for: Qualified placebo for trials of herbal medicine treatment in rare diseases? A cross-sectional analysis
Source: Orphanet J Rare Dis. 2023 Nov 30;18:373. doi: 10.1186/s13023-023-02987-w (PMC10691121; doi:10.1186/s13023-023-02987-w)
Supplement: Supplementary file 3 — Additional file 3. Summary of abstracts of studies published in Chinese. [file 13023_2023_2987_MOESM3_ESM.docx]

**Qualified Placebo for Trials of Herbal Medicine Treatment in Rare Diseases? A Cross-Sectional Analysis**

Yixuan Li^a^, Peipei Du^a^, Xuebin Zhang^a^, Chenyu Ren^a^, Xinyi Shi^a^, Xinglu Dong^a*^, Chi Zhang ^a,b*^

^a^ Dongzhimen Hospital, Beijing University of Chinese Medicine, Beijing, China

^b^ Institute for Brain Disorders, Beijing University of Chinese Medicine, Beijing, China

*** Corresponding Author:**

Chi Zhang, M.D., Ph.D., Dongzhimen Hospital, Beijing University of Chinese Medicine, 5 Haiyuncang Street, Dongcheng District, Beijing 100070, China

Tel.&Fax.: +86-10-84013209

E-mail: saga618@126.com

Xinglu Dong, M.M., Dongzhimen Hospital, Beijing University of Chinese Medicine, 5 Haiyuncang Street, Dongcheng District, Beijing 100070, China

Tel.&Fax.: +86-10-84013209

E-mail: arthasdxl@163.com

We arrange them in the order in Supplementary Material 4. Author names are highlighted in blue.

1. Clinical Efficacy and Safety Evaluation of Supplementary Sijunzi Decoction in Treatment of ALS Patients with Splenasthenic Syndrome

Xuying Zhu

Objective: To investigate the value of supplementary Sijunzi Decoction in improvement of the life functions and delay the disease progression of amyotrophic lateral sclerosis (ALS) patients with splenasthenic syndrome.

Methods: This study was designed as a randomized, double-blind and placebo-controlled clinical trial, and 50 ALS patients with splenasthenic syndrome during January 2013 and December 2015 were randomly divided into supplementary Sijunzi Decoction and placebo groups ( n = 25 for each group) . Supplementary Sijunzi Decoction group was treated with supplementary with Sijunzi Decoction on the basis of western medicine treatment, while the placebo group was treated with placebo on the basis of western medicine. The course of treatment was 9 months in the two groups. In two groups, ALS function rating score (ALSFRS-r) and ALS symptom score of TCM splenasthenic syndrome were observed and compared before treatment, 3 months, 6 months and 9 months of treatment, and 3 months after stopping medication, and incidence rates of adverse reactions during the treatment were also observed and recorded.

Results: The differences in ALSFRS-r and ALS symptom scores of TCM splenasthenic syn- drome were statistically significant after treatment between the two groups by generalized estimating equations analysis ( Wald χ2 = 594. 155, P < 0. 05; Wald χ2 = 452. 196, P < 0. 05), and especially in the TCM splenasthenic syndrome, the supplementary Sijunzi Decoction effectively delayed the deterioration of related symptoms. In the two groups, the analysis of time levels of gross and fine motors in ALSFRS-r showed statistically significant differences (Wald χ2 = 166. 976, P < 0. 05; Wald χ2 = 97. 015, P < 0. 05); however, the differences in bulbar and respiratory symptom scores were not statistically significant ( Wald χ2 = 0. 007, P > 0. 05; Wald χ2 = 1. 918, P > 0. 05). There were no serious adverse events during the treatment in the two groups.

Conclusion: Supplementary Sijunzi Decoction can delay the deterioration of neurological function in ALS patients with splenasthenic syndrome and has good clinical efficacy in gross and fine motors.

2. Clinical study on treatment of Chong meridian qi adversely ascending type amyotrophic lateral sclerosis by Shenzhe Jiangqi

Chao Pan

Objective: To assess the effects of Shenzhe Jiangqi Powder on improving the clinical symptoms of amyotrophic lateral sclerosis and enhancing the quality of life of patients by comparing its clinical efficacy for Chong meridian qi adversely ascending type ALS with Rilutek Tablets, and to explore its therapeutic mechanism.

Methods: The JVIL group administrated JI、IVEILING injection by intravenous drip and took orally simulated riluzole tablet while the control group took orally riluzole tablet and administrated simulated Jiweiling injection by intravenous drip. And 28 days was a treatment course. We observed the clinical effect with the indexes such as the clinical symptoms and signs score, evaluation quality of life, motor unit number estimation (MUNE), respiratory function, and so on.

Conclusion: The JWL injection had certain treating effects on amyotrophic lateral sclerosis and was one kind of safe and effective Chinese agent.

3. The clinical study of Jiweiling injection indealing with amyotrophic lateral sclerosis

Wenlong Ma

Objective: To observe and evaluate the clinical efficacy of Jiweiling injection in the treatment of amyotrophic lateral sclerosis (ALS). Methods: Patients with ALS were randomly divided into a Jiweiling group and a control group, with 30 cases in each group. In the Jiweiling group, JIweiling injection was given intravenously and Milirutai tablets were given orally. In the control group, Lirutai tablet was given orally and Mixihuiling injection was given intravenously. The course of treatment was 28 days. The clinical symptoms and signs score, quality of life assessment (ALSAQ-40), motor unit number estimation (UMNE) and respiratory function test were used to observe the clinical efficacy.

Results: There was no significant difference in ALSFRS, modified Norris and Appel score between the two groups before treatment (P>0.05). The ALSFRS and modified Norris scores were significantly increased after treatment in the treatment group (P>0.05). The Appel score was significantly decreased after treatment in the Jiweiling group, and there was significant difference before and after treatment (P<0.05). There were significant differences in the difference of ALSFRS, modified Norris and Appel score after treatment between the two groups (P<0.01), indicating that the clinical symptom scores of ALS patients could be improved by Jiweiling. There was no significant difference in quality-of-life score (ALSAQ-40) between the two groups before treatment (P<0.05). The quality-of-life score (ALSAQ-40) was significantly higher in the treatment group than in the control group (P<0.01). The difference of scores after treatment between the two groups was significant (P<0.01), indicating that the quality of life of patients could be improved by Jiweiling. There was no significant difference between the two groups before treatment (P>0.05); There was no significant difference in MUNE between the two groups after treatment (P>0.05), indicating that the Jiweiling group had a protective effect on motor neurons; In the control group, MUNE decreased significantly after treatment (P<0.05); The difference of IvlUNE after treatment between the two groups was significant (P<0.01), indicating that the protection of motor neurons in the Jiweiling group was better than that in the control group. VC, FVC, FEVl and MVV% were significantly increased after treatment in the Jiweiling group, and there were significant differences compared with those before treatment (P<0.01); Control group after treatment vital capacity, vital capacity, VC), forced vital capacity, forced vital capacity, FVC), the first second forced expiratory volume (forced expiratory volume, FEVl) and maximal voluntary ventilation (MVV%) were not significantly different from those before treatment (both P>0.05); In the Jiweiling group, 2 cases (6.67%) were clinically controlled, 7 cases (23.33%) were markedly effective, 19 cases (63.33%) were effective, 2 cases (6.67%) were ineffective, and the effective rate was 93.33%. The control group was 0 cases (0.00%), 2 cases (6.67%), 14 cases (46.67%), 14 cases (46.67%), and the effective rate was 53.33%. In the control group, 22 of 30 patients who took Lirutai had abnormal liver function (mainly manifested as increased transaminase). There was no adverse reaction in the Jiweiling group.

Conclusion: Jiweiling injection is a safe and effective new traditional Chinese medicine preparation for the treatment of amyotrophic lateral sclerosis (ALS).

4. A single case randomized controlled trial of Qiangji Jianli capsule in the treatment of myasthenia gravis with spleen-stomach qi deficiency

Guoyu Qiu

Objective: A single-case randomized controlled trial was conducted to study the clinical efficacy and safety of Qiangji Jianli Capsules in the treatment of myasthenia gravis due to spleen-stomach qi deficiency, providing strong clinical evidence for the treatment of myasthenia gravis by TCM syndrome differentiation, and exploring the treatment of Qiangji Jianli Capsules in the treatment of myasthenia gravis. Weak mechanism of action.

Methods: Five patients with myasthenia gravis were collected from outpatient clinics and wards who met the inclusion criteria, Osserman's classification was type IIa or type IIb, and the TCM syndrome was spleen-stomach-qi deficiency syndrome. This study used a single case randomized controlled trial. The round of drug treatment program is 8 weeks, the treatment period and the control period are each 4 weeks, the treatment sequence is random, the intermediate drug wash-out period is 1 week, the treatment period: Qiangji Jianli Capsule + Western medicine basic treatment, the control period: placebo + Western medicine basic treatment, total treatment for 29 weeks. The differences of QMG score, MGC score and MG-PRO score between the two groups before and after medication were compared and analyzed.

Results: A total of 5 subjects were included, of which 1 case fell off, and 4 cases completed the test. Compared with before treatment, QMG, MGC, and MG-PRO after treatment with Qiangjijianli Capsule were significantly improved, and the difference was statistically significant; after placebo treatment, QMG, MGC, and MG-PRO did not change significantly, and there was no statistical difference. Scientific significance: The difference between the two groups after treatment with Qiangji Jianli Capsule and placebo is statistically significant. Subject 1’s MGC and MG-PRO scores were significantly improved during the treatment period, and the difference was statistically significant (P＜0.05); Subject 4’s QMG scores during the treatment period were significantly reduced, and the difference was statistically significant (P＜0.05); The scores of the rest of the subjects during the treatment period showed an improvement trend, but the difference was not statistically significant. There were no obvious adverse drug reactions during the treatment.

Conclusion: QiangjiJianli Capsules combined with western medicine in the treatment of myasthenia gravis due to spleen-stomach qi deficiency is significantly better than the western medicine group in terms of Chinese and Western medical efficacy assessment and improvement of the quality of life of patients, and it is safe and effective. There were no adverse reactions in the study. In the individualized efficacy evaluation, the efficacy difference between subjects is obvious. Due to the different conditions of individual patients, for some cases, there is no significant difference in QMG, MGC, and MG-PRO scores between Qiangjijianli Capsule and placebo.

5. Curative Effect of Qing Ji Jian Li Capsule in a Randomized, Double-blind, Self-cross-controlled Test for Myasthenia Gravis

Tietao Deng

Ten inpatients with generalized myasthenia. gravis were treated with Qiang Ji Jian Li Capsule (QJJLC). According to the design of a randomized, double-blind, self-cross test, prednisone and placebo were used as control. The result showed that the total effective rate of QUJLC was 90%, prednisone 70% and placebo 10%. The effective rate of QJJLC was higher than that of the prednisone, but there was no significant difference between them. Yet QJJLC is considered an ideal medicine for myasthenia gravis.

6. Clinical efficacy of compound Huang Qi combined with bromopyzia in the treatment of myasthenia gravis

Jingsheng Zhang

Objective: To evaluate the clinical efficacy of compound Huang Qi combined with western medicine treatment of myasthenia gravis with spleen and kidney deficiency syndrome, and the efficacy of the syndrome of traditional Chinese medicine.

Methods: a randomized controlled trail was conducted, 248 cases of myasthenia gravis with spleen and kidney deficiency syndrome were enrolled and divided into treatment group ( 125 cases) and control group( 123 cases) ; the treatment group was given basic treatment of Western medicine combined with compound Huang Qi, control group with placebo plus Western medicine treatment, treatment for 12 weeks. The clinical efficacy of the two groups was evalua- ted by the total score of TCM symptoms. By comparing the difference between the two groups of TCM symptoms, the total score of TCM symptoms and the scores of main symptoms in 4 weeks, 8 weeks and 12 weeks after treatment, the improve- ment of TCM symptoms was evaluated.

Results: the total effective rate in the treatment group was 79. 20% , the control group was 38. 21% , and the treatment group was better than the control group ( P < 0. 05) . After treatment, 8 weeks and 12 weeks, the treatment group TCM symptoms score, main symptoms and secondary symptoms were lower than the control group ( P < 0. 05) ; after 8 weeks of treatment, the treatment group of the eyelid ptosis, dysphagia symptoms and mastica- tory score lower than the control group ( P < 0. 05) ; 12 weeks after the treatment. The treatment group of the eyelid ptosis, dysphagia, scores of symptoms and chewing fatigue, sluggish speech were lower than the control group ( P < 0. 05).

Conclusion: compound Huang Qi combined with bromopyzia treatment of myasthenia gravis with spleen and kidney defi- ciency syndrome, can significantly improve the eyelid ptosis, chewing and swallowing symptoms firstly, and significantly improve the symptoms of fatigue, sluggish speech.

7. To evaluate the efficacy of compound Huangqi granules in the treatment of myasthenia gravis of spleen-kidney deficiency type Ⅰ and Ⅱ.

Wenjun Qiao

Objective: To observe the efficacy of compound Huangqi granules in the treatment of myasthenia gravis of spleen and kidney deficiency type I and II.

Methods: A total of 248 outpatients were collected from the Affiliated Hospital of Liaoning University of Traditional Chinese Medicine, General Hospital of Shenyang Military Region and Shengjing Hospital of China Medical University. A randomized, double-blind, multicenter, placebo-controlled clinical design was used. The randomized system was from the Clinical Evaluation Center of China Academy of Traditional Chinese Medicine. The treatment group was given compound Huangqi granules + basic western medicine (pyridostigmine bromide), and the control group was given placebo. The patients were followed up for 6 months. The acquired myasthenia gravis absolute score (MGFA) was evaluated at 0 w, 4 w, 8 w, 12 w and 36 w. Serum acetylcholine receptor antibody was detected at 0 w, 12 w and 36 w.

Results: The absolute clinical scores of the treatment group were lower than those of the control group at 8 w, 12 w and 36 w, P<0.05 between the two groups. The difference between the two groups was statistically significant. P<0.05 of the treatment group at 4, 8, 12 and 36 weeks were compared with that of the control group statistically significant difference within the group. In the control group, the clinical absolute scores were compared at 12 weeks, 36 weeks and 0-week P<0.05, statistically significant difference within the group. The levels of serum acetylcholine receptor antibody in the treatment group were significantly lower than those in the control group at week 12 P<0.05, showing a significant difference.

Conclusion: Compound Huangqi granules can effectively improve the symptoms of MG patients with spleen-kidney deficiency type I and II, and the onset time is 4 weeks. It has a good improvement effect on ocular muscles and dysphagia. Compound Huangqi granules can reduce the concentration of serum acetylcholine receptor antibody.

8. Clinical and experimental study of Astragalus compound in the treatment of myasthenia gravis

Guanghua Niu

Purpose: Observe the complex recipe of Astragalus membranaceus's clinical effect in treating Myasthenia graves, provide its experimental basement; observe the influence of the complex recipe of Astragalus membranaceus to T lymphocyte, Tc1/Tc2, Thi/Th2, IFN-Y, IL-4, TNF-a, IgG. IgA, IgM, C3. C4. AchRAb in peripheral blood of myasthenia gravis patients, reveal its immunological regulation effect on the cellulax level.

9. Clinical study of Qiangji Jianli decoction in the treatment of myasthenia gravis

Zhisui Ou

Myasthenia gravis (MG) is a T cell-regulated, antibody-mediated autoimmune disease. Impaired neuromuscular transmission characteristic of MG results from antibodies against the nicotinic acetylcholine receptor (AchR) of skeletal muscle. Myasthenia Crisis is characterized by a fast, markedly decrease of muscle strength and compromise of bulbar muscles with dysphonia, dysphagia and aspiration. Traditional Chinese Medicine (TCM) consider MG belong to Lian Fei, BaoChui, TouQing, Daqixiaxian. Through compensate spleen and kidney to treat MG, Professor DengTieTao consider MG belong to XUSUN illness, involve two meaning weak and damage. The mechanism is spleen and stomach weak damage, involved five guts. He creates QiangJiJianLiYin series to treat MG and gain the good effect. Understanding the Qiangji Jianli Yin's clinic curative effect to cure Myasthenia Gravis belong to deficiency of spleen and Myasthenia Crisis and review its security. Stochastic contrast 36 patients with flaccidity-syndrome, exactly spleen flaccid, to observe the clinical curative effect of the normal regulation treatment combining strengthening the spleen treatment method (Qiangji Jianli Yin) and which improvement of myasthenia gravis and spleen flaccidity. Observed 42 patients of rescure of Myasthenia Crisis, analyzed QiangJilianLiYin's short-term and long-term curative effect, summarized experience of rescuing Myasthenia Crisis.

The result shows that the clinical curative effect comparison (P<0.05), absolute

and opposite grade comparison (P<0.05), Chinese medicine symptoms sum grad comparison (P<0.05 )and Chinese medicine symptoms comparison(P<0.05), the normal regulations treatment combining Qiangji Jianli Yin treatment method show the distinct effect.

Conclusion: to adopt the normal regulations treatment combining Qiangji Jianli Yin treatment method to treat the patient of Myasthenia Gravis belong to deficiency of spleen, not only have outstanding clinical curative effect, but also have good effect on myasthenia gravis and spleen flaccidity-syndrome. 42 patients rescured by TCM were all succeed, short-term curative effect is 100% and ling-term is 90.48%, whether side-effect or expenditure are exel comparing to modern medicine.

10. Clinical observation of Astragalus compound granules in the treatment of myasthenia gravis.

Bo Bao

Purpose：To evaluate the clinical efficacy of Engelhardtia compound granules. Material and method： 60 patients were diagnosed with spleen and kidney two deficiency type of myasthenia gravis, were from March 2014 to March 2015 in the Affiliated Hospital of Liaoning University of Traditional Chinese Medicine of patients. At the age of 14-75, 25 cases of male, female 35 cases. The 19 cases of myasthenia gravis patients, 23 cases of patients with IIA type MG, 18 cases of patients with type IIB MG. The duration of 3-48 months. The use of randomly selected patients in 1:1 group, 30 cases were divided into treatment group and control group of 30 cases. The treatment group was given Engelhardtia compound granule + pyridostigmine bromide, the control group was given placebo + pyridostigmine bromide. Treatment for 36 weeks, respectively, 4 weeks, 8 weeks, 12 weeks and 36 weeks observed two groups of patients with clinical absolute scores, total score of Chinese medicine symptom changes and TCM syndrome score changes and observe clinical efficacy of integrated traditional Chinese and Western medicine of two groups of patients. The data statistical analysis software SPSS17.0. Sample comparison of measured data, the normal distribution test, accord with normal distribution were of t test, rank sum test does not meet the normal distribution: count data chi square test, P < 0.05 for differences and is statistically significant.

11. Clinical efficacy of compound Huangqi granules in the treatment of myasthenia gravis

Bo Bao

Objective: To observe the clinical efficacy of Astragalus compound granules in the treatment of myasthenia gravis. [Methods] A total of 241 patients with myasthenia gravis were randomly divided into treatment group (n = 121) and control group (n = 120). After 12 weeks of treatment, the curative effect was observed and compared between the two groups.

Results: The absolute clinical scores and total TCM symptom scores of the two groups after treatment were lower than those before treatment (P<0.01), and the treatment group was better than the control group after 8 weeks and 12 weeks of treatment (P<0.01); In the treatment group, 2 cases were basically controlled, 47 cases were markedly effective, 46 cases were effective, and 26 cases were ineffective, with a total effective rate of 78.51%. In the control group, 0 cases were basically controlled, 8 cases were markedly effective, 16 cases were effective, and 96 cases were ineffective, with a total effective rate of 20.00%. p<0.01).

Conclusion: Astragalus membranaceus compound granules is effective in the treatment of myasthenia gravis.

12. Clinical study on treatment of myasthenia gravis with zhongjiling tablets

Fengquan XU

Objective: To study the curative effect of Zhongjiling Tablets on I A myasthenia gravis.

Methods: By the methods of randomized, double-blind, double simulated and control, the patients were divided into the treatment group (72 cases) and treated orally with Zhongjiling Tablets and prednisone placebo, and the control group (72 cases) treated orally with prednisone and Zhongjiling placebo. The symptoms, signs, the titer of serum

acetylcholine receptor antibodies, the decreasing percent of the action potential and IL-1B, sIL-2R, IL-6 of the patients in two groups were observed.

Results: The clinical effect scores of the two groups showed no significant difference (P>0.05). The treatment group had a better effect in symptom-relieving than the control group (P<0.01). There was no significant difference (P>0.0S) in the titers of the serum acetylcholine receptor antibodies and IL-1B, sIL-2R, IL-6 between two groups.

Conclusion: Zhongjiling Tables has a certain curative effect on IIA myasthenia gravis

13. The effect of Zhongjiling tablet on T lymphocyte subgroup of II A type myasthenia gravis patients and the level of INF-γ, IL-4 and TGF-β

Yiling Wu

Objective: To investigate the cellular immunological regulation mechanism of Zhongjiling tablet on the myasthenia gravis(MG) patients.

Methods: The myasthenia gravis patients were randomly divided into 2 groups the curing group and the control group 30 cases per group Patients of the curing group were administered Zhongjiling tablet and prednisone placebo patients of the control group were administered prednisone tablet and Zhongjiling tablet placebo Course of treatment was 12 weeks The distribution of T lymphocyte subgroup of myasthenia gravis patients was detected by flow cytometry: The content of INF-γ, IL-4 and TGF-β of the patient’s peripheral blood mononuclear in vitro were detected by ELISA kits.

Results: After treatment CD4^+^ T cell percentage and the ratio of CD4^+^ / CD8^+^ decreased significantly (P <0.05 ), compared with that of the control group (P <0.05). The CD4^+^ T cell percentages increased through treatment compared with that of pre-treatment there was an obvious difference (P<0.05), while the control group had no difference as well(P>0.05). The INF-γ and IL-4 and TGF-β of the treating group depressed compared with that of pretreatment significant difference exited (p<0.01). and TGF ß increased (vs pre-treatment P<0.01) after treatment while in control group INF-γ, IL-4 and TGF-β were all depressed (vs pretreatment P<0.01).

Conclusion: Zhongjiling tablet could adjust the distribution of T Lymphocyte subgroup and exudation of INF-γ, IL-4 and TGF-β, which is one of the uno logical regulation mechanisms of Zhongjiling tablet.

14. Efficacy of traditional Chinese medicine Fuyuanyi capsule combined with pyridostigmine in the treatment of 60 cases of myasthenia gravis

Xiangchun Wang

Myasthenia gravis (MG) is an acquired autoimmune disease with transmission dysfunction of the neuromuscular junction. In the past, the efficacy of western medicine alone was poor and the recurrence rate was high. In many years of clinical practice, we have explored a method of treatment with integrated traditional Chinese and western medicine, that is, treating the root cause of traditional Chinese medicine and treating the symptom of western medicine. The method of scientific combination of the two has good curative effect. This article reports the observation study of 60 cases from 2007 to 2008.

15. The Clinical Effect of Jianjining Granule on the Myasthenia Gravis Patients with Spleen-kidney Deficiency Type

Chao Jiang

Objective: To investigate the interventional effect of Jianji Ning Granule on the myasthenia gravis (MG) patients with spleen-kidney deficiency type.

Methods: Patients were randomly divided into two groups. Control group were treated with basic western medicine(prednisone or olfactory pyridostigmine), while experimental group were treated with basic western medicine combined with Chinese medicine. The efficacy was compared and the Quantitative score of myasthenia gravis (QMG) was measured before and after treatment 1、3、6 months.

Results: After treatment all the patients were improved, while the total effective rate after 3 and 6 months in the experimental group were significantly higher than those in the control groups.

Conclusion: Therapy with Jianji Ning Granule combined with Western medicine shows definite effects in treating MG patients with spleen-kidney deficiency type, especially shows more significantly after 6 months.

16. Clinical Efficacyy of Yiqi Chushi Recipe in Treating Myasthenia Gravis

Xiaoping Shuang

Objective: To observe the efficacy of Yiqi Chushi Recipe (YCR)in treating myasthenia gravis (MG)patients and its effects on the expression of PD -1/PD -L1.

Methods: 38 patients type with I and II MG from clinics and wards of the Affiliated Hospital of Hubei University of Traditional Chinese Medicine from October 2012 to November 2013 were randomly divided into the treatment group (20 cases) and the control group (18 cases). Patients in the treatment group were treated by YCR with basic Western medicine(prednisone or olfactory pyridostigmine), while those in the control group were only treated by basic Western medicine. The duration of treatment was ten weeks. The efficacy was evaluated by the quantitative score of myasthenia gravis (QMG)and the levels of PD-1/PD -L1 expression on monocytes were measured by flow cytometry before and after treatment in both groups. Meanwhile, the safety evaluation was performed.

Results: The significant effective rate of the treatment group was 70.0% whereas 45.0% in the control group, and there was a significant difference (P<0.05).The total effective rate of the treatment group was 90.0% whereas 89.0% in the control group, and there was no significant difference (P >0.05). Compared with the same group before treatment, the levels of PD -1/PD -expression decreased significantly in the treatment group (P<0.05). There was no obvious change in each index of the control group after treatment (P >0.05). Compared with the control group after treatment, the peripheral blood levels of PD -1/ PD -L1 expression decreased in the treatment group, showing statistical differences (P <0.05). During the course of treatment, there was one patient in the control group and one patient in the treatment group with mild diarrhea, nausea, and vomiting.

Conclusion: YCR could significantly improve clinical symptoms of patients with MG, and decrease the levels of PD -1/PD -L1 expression, with no obvious adverse reaction.

17. Clinical observation of Shenmai injection in the treatment of myasthenia gravis

Jinbo Lou

Abstract: Objective: To observe the clinical efficacy of Shenmai injection in the treatment of myasthenia gravis. Methods: A total of 70 patients with myasthenia gravis were randomly divided into a treatment group (36 cases) and a control group (34 cases). Both groups were treated with pyrimethamine bromide and prednisone. The treatment was given once a day for 15 consecutive days, and the clinical efficacy of the two groups was compared. Results: After treatment, the clinical evaluations of the two groups of patients were significantly lower than those before treatment (P<0.01), and the improvement in the treatment group was significantly better than that in the control group (P<0.05); The overall response rate was 94.4% in the treatment group and 94.1 % in the control group. The difference between the two groups was not statistically significant (P<0.05). Conclusion: Shenmai injection is effective in the treatment of myasthenia gravis, which is worthy of clinical application

18. The Treatment of Idiopathic Pulmonary Fibrosis about Anti-fiber Particles and Its Effects on T lymphocyte subpopulation

Xinyu Bao

Objective: The topic under the guidance of TCM theory, clinical and laboratory instructors in the past based on the research, study pulmonary fibrosis pathogenesis, Qi and Yin with a toxin, the role of Huatan Tongluo Kangxian particle treatment of pulmonary fibrosis. After treatment of patients by improving the degree of symptoms and signs, on lung function, lung compliance, blood gas analysis, CT of the chest and changes of T lymphocyte subpopulation levels before and after treatment the changes in. Reveal the mechanism of effect of Chinese medicine, for the objective study of Chinese medicine and the future of Chinese medicine treatment of pulmonary fibrosis drug laws seek to lay the groundwork for the treatment of pulmonary fibrosis open up new avenues.

Method: According to the order before and after admission patients were randomly divided into two groups: treatment group and control group, 30 cases each. Two groups of patients according to disease and sputum culture conditions, give antibiotics, controlled oxygen therapy and other therapeutic measures. Before treatment and observation period 15 not to use corticosteroids. The treatment group taking anti-fiber particles, while with a small dose of prednisone, for 3 months; The control group was treated with prednisone, for 3 months. Both groups give the same nutrition therapy, avoid the use of immunosuppressive agents and Traditional Chinese medicine preparations. Addition to disease progression during treatment, death to stop observation, not free to change the treatment.

Conclusion: Anti-fiber particles can significantly improve symptoms and signs in patients with pulmonary fibrosis, to improve pulmonary function and PaO2, Increase lung diffusing capacity and improve the chest HRCT, to effectively improve the quality of life of patients and can delay the disease's further development. Anti-fiber particles could improve cellular immunity and humoral immunity index, inhibit profibrotic cytokine production and expression, form against pulmonary fibrosis. Anti-fiber particles can reduce drug side effects, improve patient physique.

19. Clinical Study of Luotong Xianrong Yin in Treatment of Idiopathic Pulmonary Fibrosis

Zhaoyi Fang

Objective: To study the effects of Luotong Xianrong Yin on the scores of TCM syndromes, dyspnea and quality of life in patients with idiopathic pulmonary fibrosis (IPF).

Methods: 120 patients with IPF were randomly divided into observation group (60 cases) and control group (60 cases) . Both groups were given routine medical treatment and nursing. Patients in the observation group were given Luotong Xianrong Yin additionally, while those in the control group were given placebos. After 12 weeks, the scores of TCM syndromes, dyspnea and quality of life were observed before and after treatment.

Results: Compared with the scores before treatment, the scores of TCM syndromes and dyspnea in both groups decreased significantly after treatment, and the scores of quality of life improved significantly (P < 0. 05) ; compared with the control group, the improvement of the above indexes in the observation group was also significant (P < 0. 05).

Conclusion: Luotong Xianrong Yin can improve the clinical symptoms and quality of life of patients.

20. Clinical study of Fuzheng Tongluo decoction in the treatment of idiopathic pulmonary interstitial fibrosis syndrome of lung and kidney qi deficiency

Qi Liu

Objective: To evaluate the efficacy of Fuzheng Tongluo Recipe in the treatment of lungandkidny Qideficiencyof idiopathic pulmonary fibrosis.

Methods: According to the principle of randomized, double-blind, placebo, and parallel control, 68 patients with idiopathic pulmonary fibrosis, pulmonary qi-deficiency syndrome, and non-acute aggravation were included. The treatment group was treated with non-drug therapy and Fuzheng Tongluo Granule. Treatment, control group Wester medicine non-drug treatment plus Chinese medicine placebo, observation record for 16 weeks.

Conclusion: Western medicine combined with traditional Chinese medicine Fuzheng Tongluo Recipe in the treatment of patients with non-acute aggravation of IPF pulmonary-kidney qi deficiency syndrome can improve some clinical symptoms and syndromes of patients, may delay the trend of lung function and imaging deterioration, improve patient exercise endurance Trends to improve the quality of life of patients.

21. Effects of Qizhu Kangxian Granules on the pulmonary function and life quality in patients with idiopathic pulmonary fibrosis

Sijia Guo

Objective: To observe the effects of Qizhu Kangxian Granules on the pulmonary function and life quality in patients with idiopathic pulmonary fibrosis (IPF).

Methods: A total of 130 IPF patients from 6 hospitals were enrolled and assigned to two groups by stratified block randomization, 65 in the treatment group (TG) and 65 in the control group (CG). All the patients received disease education, health condition follow-up and family oxygen therapy. Additionally, the patients in the TG were treated with Qizhu Kangxian Granule, while the patients in the CG were treated with Qizhu Kangxian Granule placebo, 8g/ package, 2 packages each time, 2 times per day. The therapeutic course for both groups was 48 weeks. The pulmonary function tests including forced vital capacity (FVC), predicted FVC (FVC%), variation of FVC before and after treatment (ΔFVC), carbon monoxide diffusing capacity (DLCO) and predicted DLCO (DLCO%), the scores of St. George’s Respiratory Questionnaire (SGRQ) and traditional Chinese medicine clinical symptoms (TCMCS) of the patients were observed. Moreover, we calculated the change in FVC between before and after treatment (ΔFVC).

Results: The total effective rate in the TG was 50.77% and was superior to that in the CG (10.77%) (*P*<0.05). Compared with before treatment within the groups, the FVC, FVC%, DLCO and DLCO% of the two groups decreased significantly after treatment (*P*<0.05). After treatment, the FVC, FVC%, DLCO and DLCO% of the TG were higher than those in the CG (*P*<0.05), while the ΔFVC of the TG was lower than it in the CG (*P*<0.05). Comparing with before treatment within the group, the scores of SGRQ (including the scores of respiratory symptoms, disease affect and total score) and TCMCS in the TG decreased significantly after treatment (*P*<0.05), while those in the CG increased significantly (*P*<0.05). Comparing the two groups after treatment, the scores of SGRQ (including the scores of respiratory symptoms, disease affect and total score) and TCMCS in the TG were lower than those in the CG (*P*<0.05).

Conclusion: Qizhu Kangxian Granules might effectively treat the IPF patients by ameliorating the clinical symptoms, protecting the pulmonary function from deterioration and improving the life quality, which is worth being explored deeply.

22. Clinical Effectiveness on Feiwei Medicinal Instant Granules in Treatment of Patients with Idiopathic Pulmonary Fibrosis and Its Effect on Coagulation Function

Ning li

Objective: To observe clinical effectiveness of Feiwei medicinal instant granules in treatment of idiopathic pulmonary fibrosis (IPF) patients with insufficiency of QI of the lung and kidney and blood stasis and its effect on coagulation function. Methods A total of 50 IPF patients with insufficiency of QI of the lung and kidney and blood stasis during July 2014 and July 2017 were randomly divided into observation group (n = 25) and control group (n = 25) ac- cording therapeutic methods. All patients received conventional therapy, and then observation group was treated with Feiwei medicinal instant granules orally, while control group was added with placebo orally, and the course of treatment was 12 weeks. Changes of dyspnea scores, lung function, TCM scores and coagulation function before and after treatment were observed in two groups. Results The overall response rates of TCM syndromes in observation group were significantly higher than those in control group (P < 0. 05). After treatment, in observation group, gasping, anergy, dyspnea scores and fibrinogen level were significantly lower (P < 0. 05) , while values of percent of actual to predicting detection values in diffusion capacity of carbon monoxide and six-minutes walking test were significantly higher than those before treatment and in control group ( P < 0. 05).

Conclusion Feiwei medicinal instant granules in treatment of IPF patients with insufficiency of QI of the lung and kidney and blood stasis may effectively relieve dyspnea symptom and improve lung function so as to improve quality of life and coagulation function.

23. Clinical observation of Fuzheng Tixie Souluo Formula in the treatment of idiopathic pulmonary fibrosis

Lanfeng Cui

Objective To observe the clinical effect and safety of Fuzheng Tixie Souluo Formula in trea- ting idiopathic pulmonary fibrosis (IPF). Methods Randomized double-blind placebo-controlled study was used in this study. 76 patients with mild and moderate IPF were randomly divided into the treatment group and control group, with 38 in each. The treatment group was given Fuzheng Tixie Souluo Formula orally, while the control group was given placebo orally. The treatment lasted 12 weeks. Pulmonary function and HRCT were examined before and after treatment. Symptoms and signs were recorded as well. CRP scores were calculated for the assessment of disease severity.

Results Comprehensive effect of the treatment group is superior to control group (P < 0. 05). Compared to the control group, symptoms of the treatment group such as shortness of breath, coughing, wheezing and dyspnea were significantly improved (P < 0. 05) , and the diffusion function was improved as well(P < 0. 05) . No reverse effects were found in both groups.

Conclusion Fuzheng Tixie Souluo Formula has positive effect on idiopathic pulmonary fibrosis.

24. Clinical study on Modified Xiayuxue Granule combined with prednisone acetate tablets in the treatment of idiopathic pulmonary fibrosis with syndromes of deficiency of qi and yin and obstruction of lung collaterals

Zhe Zhe

Objective: To evaluate the clinical efficacy of Modified Xiayuxue Granule combined with prednisone acetate tablets in the treatment of idiopathic pulmonary fibrosis (IPF) with syndromes of deficiency of qi and yin and obstruction of lung collaterals and provide the clinical evidence for the treatment of IPF.

Methods: Sixty IPF patients with syndromes of deficiency of qi and yin and obstruction of lung collaterals were included and randomly divided into the treatment group and the control group, with 30 cases in each group. The patients in the treatment group were orally treated with prednisone acetate tablets combined with Modified Xiayuxue Granule, and the patients in the control group were orally treated with prednisone acetate tablets combined with Modified Xiayuxue Granule placebo, with a course of 12 months. Before and after treatment, the scores of high-resolution CT (HRCT) and Chinese medical syndrome, the 6-minute walking distance (6MWD) and the dosage of prednisone acetate tablets in both groups were evaluated and compared.

Results: In the course of treatment, 2 patients in the treatment group and 4 patients in the control group were removed or fell off, and finally 28 patients in the treatment group and 26 patients in the control group were included for statistical analysis. Before and after treatment, there was no statistically significant difference on the HRCT score between the two groups (P>0.05). After treatment, the score of Chinese medical syndrome in the treatment group was decreased compared with that before treatment (P<0.05), but there was no statistically significant difference on the score of Chinese medical syndrome in the control group between treatment before and after (P>0.05), and the score of the treatment group was lower than that of the control group ( P<0.05). 3After treatment, the 6MWD of the treatment group was obviously prolonged compared with that before treatment (P<0.05), but there was no statistically significant difference on the 6MWD in the control group between treatment before and after (P>0.05), and the 6MWD of the treatment group was longer than that of the control group ( P<0.05). 4 There was a statistically significant difference on the dosage of prednisone acetate tablets between the two groups (P<0.05), and the dosage of prednisone acetate tablets in the treatment group was less than that in the control group.

Conclusion: Modified Xiayuxue Granule combined with prednisone acetate tablets can effectively improve the clinical symptoms of IPF patients with syndromes of deficiency of qi and yin and obstruction of lung collaterals, reduce the dosage of prednisone acetate tablets, and delay the progression of pulmonary fibrosis to some extent.

25. Clinical Study of Fuzheng Tongluo Decoction in the Treatment of Pulmonary and Kidney Qi Deficiency Syndrome of Idiopathic Pulmonary Fibrosis

Yanrong Gao

Objective: To evaluate the clinical efficacy of Fuzheng Tongluo Decoction in the treatment of idiopathic pulmonary fibrosis and deficiency of lung and kidney Qi.

Methods: According to the principle of randomization, double-blind, placebo and parallel control, 68 patients with idiopathic pulmonary fibrosis and pulmonary and kidney Qi deficiency syndrome were included. The treatment group was treated with non-drug treatment combined with Fuzheng Tongluo Granules, and the control group was treated with non- drug treatment and traditional Chinese medicine placebo. Observation records were recorded for 16 weeks.

Results: (1)Curative effect of TCM diseases and syndromes: After treatment, the total effective rate was 76.67% in the treatment group and 38.71% in the control group, with statistically significant difference (P<0.000).(2)TCM main symptom score: In the treatment group, the score of cough was significantly lower than that before treatment(P<0.05), and the scores of expectoration, wheezing, wheezing and shortness of breath were significantly lower than that before treatment(P>0.05).(3)TCM syndrome score: There were statistically significant differences between the treatment group and the TCM syndrome score group(P<0.01), but there were no statistically significant differences between the treatment group and the control group(P>0.05).(4)Pulmonary function index:FEV1/FVC decreased in the treatment group, while FVC%, DLCO% and TLC% increase in the treatment group, but there was no significant difference compared with the control group(P>0.05).(5)In terms of 6MWD:The treatment group was higher than before, but there was no statistical difference(P>0.05);the control group was lower than before, and the difference was statistically significant(P<0.05);the comparison between the two groups showed no statistical difference(P>0.05).(6)Dyspnea index(mMRC)score: No significant change was observed in the treatment group, showing no statistical difference(P>0.05);the score of the control group was lower than that before, showing a statistically significant difference(P<0.05);the comparison between the two groups showed no statistical difference(P>0.05).(7)SGRQ scale: There were statistically significant differences between the two groups in symptom manifestation, activity limitation and psychological status(P<0.05).

Conclusion: Western medicine basic treatment combined with traditional Chinese medicine Fuzheng Tongluo Decoction can improve some clinical symptoms and syndromes of IPF patients with non-acute exacerbation of lung and kidney Qi deficiency, which may delay the deterioration of lung function, improve the trend of exercise endurance and improve the quality of life of the patients.

26. Clinical Trial of Feitong Oral Liquid in Treating Idiopathic Pulmonary Fibrosis

Maorong Fan

Objective: To evaluate the efficacy and safety of Feitong oral liquid in the treatment of idiopathic pulmonary fibrosis with deficiency of qi and Yin combined with obstruction of phlegm and blood stasis.

Method: A randomized, double-blind, double-dummy, positive drug parallel control, multi-center, non-inferiority clinical trial was conducted. A total of 253 subjects were enrolled and randomly divided into high-dose Feitong group (n = 84), low-dose Feitong group (n = 85) and control group (n = 84). To observe the efficacy and safety of Feitong oral liquid in the treatment of idiopathic pulmonary fibrosis.

Results: In the comparison of curative effect, the high-dose Feitong group was not inferior to the control group. The quality of life in the high-dose Feitong group was significantly better than that in the control group (P<0.05); Blood gas analysis (arterial partial pressure of oxygen, arterial oxygen saturation, alveolar-arterial oxygen difference) and pulmonary function test were performed. The total lung capacity, carbon monoxide diffusing capacity and other indicators of the high-dose Feitong group were better than those of the control group and the low-dose Feitong group, but the difference was not significant. The incidence of adverse reactions in the high-dose and low-dose Feitong groups was significantly lower than that in the control group.

Conclusion: Feitong oral liquid is safe and effective in the treatment of idiopathic pulmonary fibrosis (both qi and Yin deficiency and phlegm and blood stasis obstruction).

27. Clinical efficacy observation of Bushen Yisui Capsule on relapsing remitting multiple

sclerosis with syndrome of deficiency of kidney-liver yin

Yongping Fan

Objective: To observe the clinical efficacy of Bushen Yisui Capsule (BSYSC) on relapsing remitting multiple sclerosis (RRMS) during remission.

Methods: The randomized, double-blind, parallel control design was carried out in this study. Sixty RRMS patients during remission were enrolled at Department of Traditional Chinese Medicine, Beijing Tiantan Hospital, Capital Medical University during 12, 2011 to 06, 2014. All the patients (treatment group, 30 cases; placebo group, 30 cases) belonged to syndrome of deficiency of kidney-liver yin and were taking orally prednisone. The treatment group taken with BSYSC for 3 months and the placebo group with placebo instead. The clinical effects were valued by expanded disability status scale (EDSS) and traditional Chinese medicine symptom scores (TCMSS).

Results: The difference of EDSS and TCMSS between two groups before treatment was not significant. Compared with pre-treatment, the EDSS score and TCMSS of treatment group and placebo group decreased significantly (*P*<0.05, *P*<0.01), and the treatment group was superior to the placebo group (*P*<0.05). The functions were improved in lassitude, irritability, blurred vision, feeling, pain, night sweat and other symptoms of treatment group (*P*<0.01, *P*<0.05), and the dinical effective rate was significantly higher than that of the placebo group (*P*<0.01).

Conclusion: The symptoms of RRMS can be alleviated by BSYSC treatment, it can improve the quality of ilfe, and it’s worthy of clinical promotion.

28. Therapeutic effect of integrated traditional Chinese and western medicine on multiple sclerosis

Yanshan Zhuang

Objective: To observe the effect of integrated traditional Chinese and western medicine in the treatment of multiple sclerosis.

Methods: Thirty patients were randomly divided into a treatment group and a control group, with 30 cases in each group. Both groups were treated with prednisone, the treatment group was treated with Buyang Huanwu decoction, and the control group was treated with scientific research protocol.

Results: The therapeutic effect of the treatment group was better than that of the control group (P<0.01).

Conclusion: Western medicine combined with Buyang Huanwu decoction has a good effect in the treatment of multiple sclerosis, which can reduce the clinical symptoms, improve the quality of life, reduce the dosage of hormone and reduce the adverse reactions of hormone therapy.

29. Clinical study of Dihuang mixture (capsule) in the treatment of acute relapsing multiple sclerosis

Mucan Lin

In this study, the Kurtzke's extended Disability Scale (EDSS) and daily life index (Barthel index) of 60 patients with acute recurrent multiple sclerosis were studied. According to the regional red randomization method, they were divided into treatment group (306) and control group (300). The treatment group was given conventional western medicine treatment based on methylprednisolone and prednisone plus Dihuang mixture (Jiaotong), and the control group was given western medicine treatment plus placebo. The results showed that there was no significant difference in Kurtzke's Expanded Disability Scale (EDSS) score between the two groups after 3 weeks of treatment, but there was significant difference in EDSS score between the two groups at 6 weeks of treatment (P ≤0.001). The Bartbel index at the 6th week after treatment was significantly different between the two groups (P < 0.05). The patients in the western group were followed up for one year to observe the recurrence times, and the comparison between the two groups was statistically significant (P<0.05). Conclusion: In the acute attack of multiple sclerosis, the conventional treatment of western medicine with hormone can quickly relieve the condition. After 6 weeks of application, the clinical effect of the treatment with Dihuang mixture (capsule) is more significant, and it is worthy of clinical promotion. Compared with the control group, the patients in the treatment group who were treated with Dihuang mixture (Jiaochu) had fewer cases of the symptoms of Yin deficiency, heat, insomnia, night sweat, dry mouth and dry throat caused by the use of hormone, and the difference was statistically significant (P<0.05)

30. Effect of Shenzhe Zhuyun mixture on esophageal lesions of systemic sclerosis

Gang Guo

To investigate the clinical effect of Shenzhe Zhurun mixture on esophageal lesions in patients with systemic sclerosis (SSc). A total of 64 patients who met the inclusion criteria were randomly divided into treatment group (cisapride tablets + cisapride tablets) and control group (cisapride tablets + Cisapride tablets). After 2 months of treatment, the clinical efficacy, esophageal disease intersection syndrome score, the barium esophageal transit time in standing and lying positions, the barium emptying index in lying position, the widest transverse diameter of esophageal dilatation, and the impact on the quality of life of patients were observed. The total effective rate was 96.88% in the treatment group and 56.25% in the control group. There was a significant difference between the two groups (P<0.01); The symptoms of esophageal lesions were significantly improved after treatment in both groups (P<0.01), and the treatment group was better than the control group (the difference between the two groups was P<0.01); The treatment group was in the opposite, supine position when barium was passed through the esophagus

The barium emptying index (P<0.01), and there was no significant change in esophageal transverse diameter. In the control group, there was a change in the transit time of barium in the supine position (P<0.01), but the barium transit time in standing position, the barium emptying index in supine position and the transverse diameter of esophagus had no significant improvement. The quality of life in the treatment group was significantly lower than that in the control group (P<0.01), but not in the control group. The results indicate that Shenzhe Zhurun mixture can enhance the esophageal motility function of patients with SSc, and it is an effective traditional Chinese medicine compound for the treatment of esophageal lesions in SSc.
