# Supplementary material for: Qualified placebo for trials of herbal medicine treatment in rare diseases? A cross-sectional analysis
Source: Orphanet J Rare Dis. 2023 Nov 30;18:373. doi: 10.1186/s13023-023-02987-w (PMC10691121; doi:10.1186/s13023-023-02987-w)
Supplement: Supplementary file 4 — Additional file 4. Chi-squared analysis. [file 13023_2023_2987_MOESM4_ESM.docx]

Supplementary Material 2

**Qualified Placebo for Trials of Herbal Medicine Treatment in Rare Diseases? A Cross-Sectional Analysis**

**Yixuan Li, Peipei Du, Xuebin Zhang, Chenyu Ren, XinYi Shi, XingLu Dong^*^, Chi Zhang^*^**

*** Correspondence:**

Chi Zhang

saga618@126.com

Xinglu Dong

arthasdxl@163.com

Chi-squared test results

|  | **Reporting items﹥7 (N=21)** | **Reporting items ≤ 7 (N=29)** | **Odds ratio (95%CI)** | **P value** |
| --- | --- | --- | --- | --- |
| **HM-intervention ^a^** |  |  |  | P=0.98 |
| Single herbal | 5 (41.7%) | 7 (58.3%) | 0.98 (0.26-3.66) |  |
| HM- formula | 16 (42.1%) | 22 (57.9%) | Reference |  |
| **Ethics approval ^b^** |  |  |  | P=0.18 |
| Yes | 12 (52.2%) | 11 (47.8%) | 2.18 (0.70-6.85) |  |
| Not reported | 9 (33.3%) | 18 (66.7%) | Reference |  |
| **Inform consent ^c^** |  |  |  | P=0.18 |
| Yes | 17 (43.6%) | 22 (56.4%) | 1.35 (0.34-5.39) |  |
| Not reported | 4 (36.4%) | 7 (63.6%) | Reference |  |
| **Sample size ^d^** |  |  |  | P=0.63 |
| 1-84 | 13 (44.8%) | 16 (55.2%) | 1.32 (0.42-4.15) |  |
| 84-150 | 8 (38.1%) | 13 (61.9%) | Reference |  |
| **Administration time ^e^** |  |  |  | P=0.89 |
| ≤1month | 12 (42.9%) | 16 (57.1%) | 1.08 (0.35-3.36) |  |
| > 1month | 9 (40.9%) | 13 (59.1%) | Reference |  |
| **Funding ^f^** |  |  |  | P=0.91 |
| non-business | 4 (16.7%) | 20 (83.3%) | 1.53 (0.31-7.69) |  |
| business or not reporting | 3 (11.5%) | 23 (88.5%) | Reference |  |
| **Therapy ^g^** |  |  |  | P=0.63 |
| add-on | 13 (44.8%) | 16 (55.2%) | 1.32 (0.42-4.15) |  |
| only placebo | 8 (38.1%) | 13 (61.9%) | Reference |  |

Table s2. Characteristics associated with relative-high quality reporting (items >7).

^a^ HM-intervention was dichotomized as singer herbal versus HM formula.

^b^ Ethics approval was dichotomized as reported versus not reported.

^c^ Inform consent was dichotomized as reported versus not reported.

^d^ The Sample size was dichotomized as 1-84 versus 84-150 according to the median value.

^e^ Administration time of placebos was dichotomized as ≤ 1 month versus > 1 month according to the median value.

^f^ Funding was dichotomized as non-business versus business involvement or not reporting.

^g^ The controlled treatment method was dichotomized as placebo added to other treatments versus only placebo.

Table s3. Characteristics associated with relative-high quality reporting (items >8).

|  | **Reporting items﹥8 (N=14)** | **Reporting items ≤ 8 (N=36)** | **Odds ratio (95%CI)** | **P value** |
| --- | --- | --- | --- | --- |
| **HM-intervention ^a^** |  |  |  | P=1.00 |
| Single herbal | 3 (25.0%) | 9 (75.0%) | 0.49 (0.052-4.49) |  |
| HM- formula | 11 (28.9%) | 27 (71.1%) | Reference |  |
| **Ethics approval ^b^** |  |  |  | P=0.11 |
| Yes | 9 (39.1%) | 14 (60.9%) | 2.83 (0.79-10.20) |  |
| Not reported | 5 (18.5%) | 22 (81.5%) | Reference |  |
| **Inform consent ^c^** |  |  |  | P=0.23 |
| Yes | 13 (33.3%) | 36 (66.7%) | 5.00 (0.58-43.49) |  |
| Not reported | 1 (9.1%) | 10 (90.9%) | Reference |  |
| **Sample size ^d^** |  |  |  | P=0.57 |
| 1-84 | 9 (31.0%) | 20 (69.0%) | 1.44 (0.40-5.16) |  |
| 84-150 | 5 (23.8%) | 16 (76.2%) | Reference |  |
| **Administration time ^e^** |  |  |  | P=0.59 |
| ≤1month | 7 (25.0%) | 21 (75.0%) | 0.71 (0.21-2.47) |  |
| > 1month | 7 (31.8%) | 15 (68.2%) | Reference |  |
| **Funding ^f^** |  |  |  | P=0.42 |
| non-business | 8 (33.3%) | 16 (66.7%) | 1.67 (0.48-5.80) |  |
| business or not reporting | 6 (23.1%) | 20 (76.9%) | Reference |  |
| **Therapy ^g^** |  |  |  | P=0.23 |
| add-on | 10 (34.5%) | 19 (65.5%) | 2.24 (0.60-8.47) |  |
| only placebo | 4 (19.0%) | 17 (81.0%) | Reference |  |

^a^ HM-intervention was dichotomized as singer herbal versus HM formula.

^b^ Ethics approval was dichotomized as reported versus not reported.

^c^ Inform consent was dichotomized as reported versus not reported.

^d^ The Sample size was dichotomized as 1-84 versus 84-150 according to the median value.

^e^ Administration time of placebos was dichotomized as ≤ 1 month versus > 1 month according to the median value.

^f^ Funding was dichotomized as non-business versus business involvement or not reporting.

^g^ The controlled treatment method was dichotomized as placebo added to other treatments versus only placebo.
