# Supplementary material for: Qualified placebo for trials of herbal medicine treatment in rare diseases? A cross-sectional analysis
Source: Orphanet J Rare Dis. 2023 Nov 30;18:373. doi: 10.1186/s13023-023-02987-w (PMC10691121; doi:10.1186/s13023-023-02987-w)
Supplement: Supplementary file 6 — Additional file 6. Risk of bias of included studies. [file 13023_2023_2987_MOESM6_ESM.docx]

**Qualified Placebo for Trials of Herbal Medicine Treatment in Rare Diseases? A Cross-Sectional Analysis**

Yixuan Li^a^, Peipei Du^a^, Xuebin Zhang^a^, Chenyu Ren^a^, Xinyi Shi^a^, Xinglu Dong^a*^, Chi Zhang ^a,b*^

^a^ Dongzhimen Hospital, Beijing University of Chinese Medicine, Beijing, China

^b^ Institute for Brain Disorders, Beijing University of Chinese Medicine, Beijing, China

*** Corresponding Author:**

Chi Zhang, M.D., Ph.D., Dongzhimen Hospital, Beijing University of Chinese Medicine, 5 Haiyuncang Street, Dongcheng District, Beijing 100070, China

Tel.&Fax.: +86-10-84013209

E-mail: saga618@126.com

Xinglu Dong, M.M., Dongzhimen Hospital, Beijing University of Chinese Medicine, 5 Haiyuncang Street, Dongcheng District, Beijing 100070, China

Tel.&Fax.: +86-10-84013209

E-mail: arthasdxl@163.com


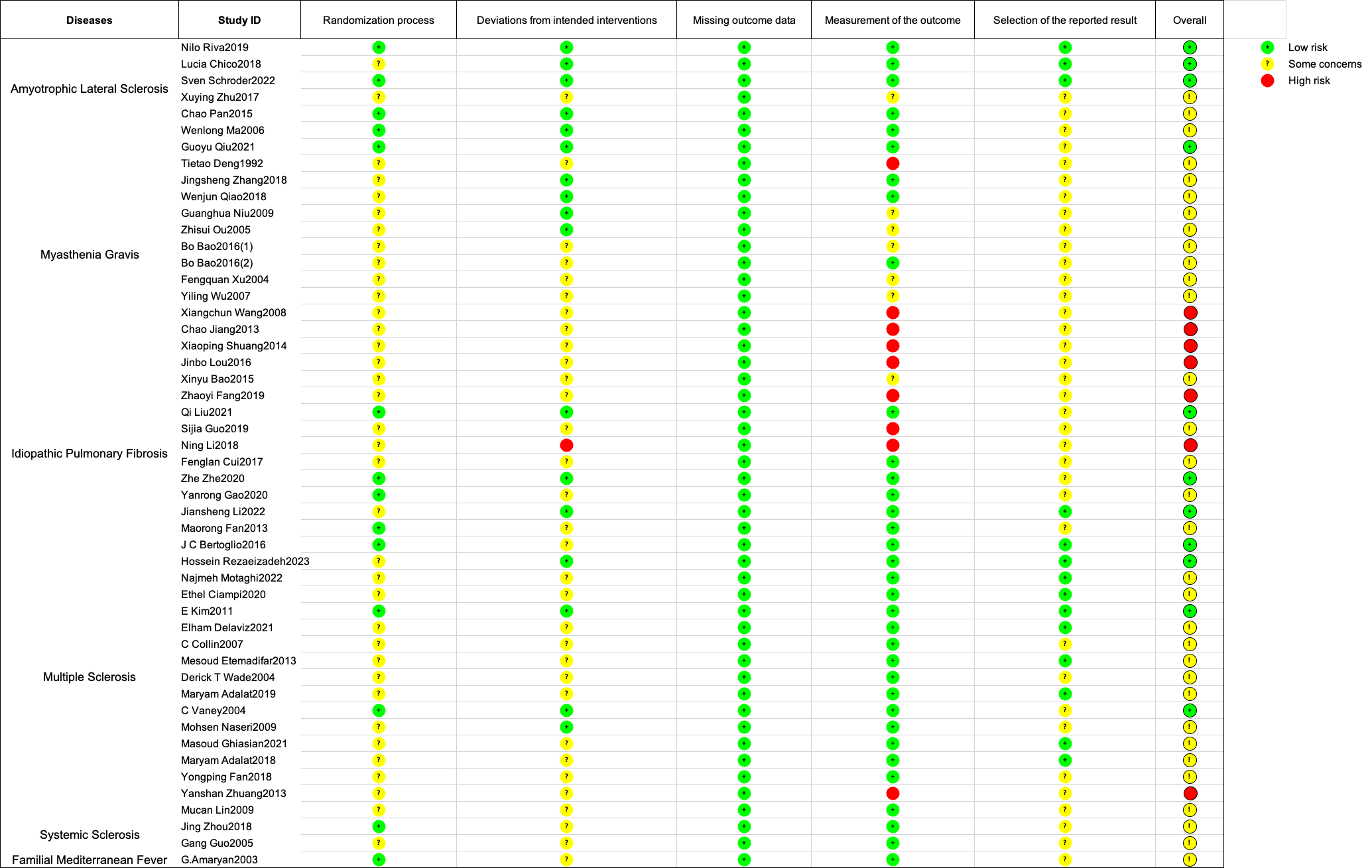


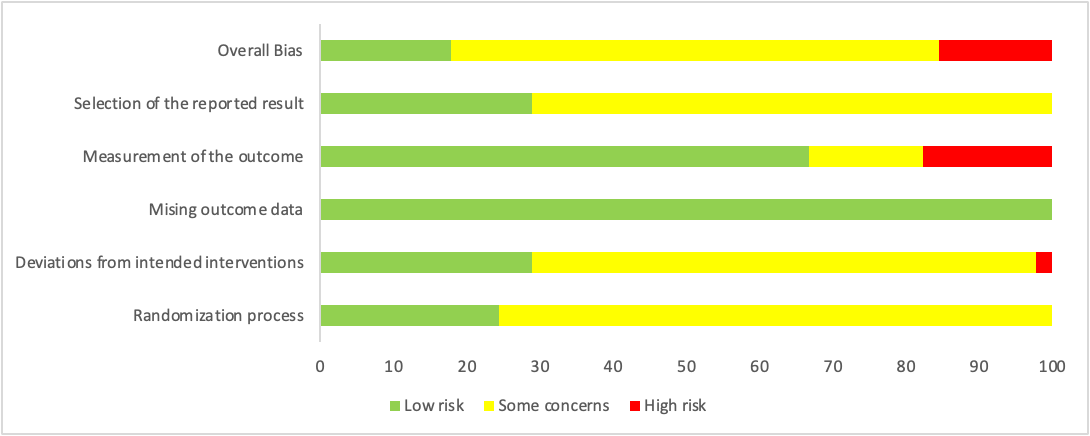


Figure S1: Risk of bias of included studies. (5 non-RCT trials and 5 trials that did not contain complete information were excluded)
